# Tuning and clinical application of large language models in Traditional Chinese Medicine: scoping review

**DOI:** 10.1186/s13020-026-01346-8

**Published:** 2026-02-19

**Authors:** Changxiao Han, Guangyi Yang, Hongtao Li, Liguo Zhu, Minshan Feng

**Affiliations:** 1 Wangjing Hospital of China Academy of Chinese Medical Sciences, Beijing, 100102 China; 2 Beijing Key Laboratory of Digital Intelligence Traditional Chinese Medicine for Preventing and Treating Degenerative Bone and Joint Diseases, Beijing, 100102 China; 3 Beijing University of Chinese Medicine, Beijing, 100029 China

**Keywords:** Traditional Chinese Medicine, Large language models, Scoping review, Clinical application, Benchmark

## Abstract

**Background and objective:**

Large Language Models (LLMs) show significant potential in healthcare, but their application in Traditional Chinese Medicine (TCM) lacks systematic evaluation. This study aims to comprehensively review LLMs tuning techniques, data construction strategies, evaluation methods, and application scenarios in TCM clinical practice.

**Methods:**

A scoping review following PRISMA-ScR guidelines was conducted. Researchers systematically searched seven databases for relevant studies published between database inception to May 2025. The analysis focused on identifying model characteristics, tuning techniques, data sources, evaluation methods, application domains and performance limitations to assess the current state and future directions of TCM-oriented LLMs.

**Results:**

We included 27 studies (21 in English, 6 in Chinese). Application domains comprised TCM knowledge consultation (10 studies) and diagnostic assistance (13 studies), with 4 studies establishing TCM LLMs evaluation benchmarks. LoRA fine-tuning was most widely used (65.2%), often combined with prompt engineering (47.8%), continued pre-training (43.5%), and retrieval-augmented generation (39.1%). Most studies (87.0%) employed multiple technique combinations. Training data balanced theoretical knowledge (classics) with clinical experience (case records), though multimodal data remained severely insufficient. Evaluation methods were multidimensional, with accuracy (63.0%) and human assessment (77.8%) most frequently used. Specialized TCM evaluation benchmarks were gradually established. Current models excel at integrating heterogeneous knowledge, basic syndrome differentiation reasoning, and cross-language knowledge conversion, but show limitations in simulating complex TCM reasoning processes and individualized diagnosis.

**Conclusion:**

Although TCM-oriented LLMs demonstrate effectiveness in knowledge consultation and diagnostic tasks, they face significant challenges in capturing TCM's holistic paradigm, data quality, and clinical evaluation. Future research should develop TCM-compatible model architectures, build standardized multimodal data ecosystems, strengthen clinical translation, and create evaluation frameworks that reflect TCM's diagnostic process.

**Supplementary Information:**

The online version contains supplementary material available at 10.1186/s13020-026-01346-8.

## Introduction

Large Language Models (LLMs) represent a significant breakthrough in artificial intelligence technology and are rapidly reshaping knowledge processing and application methods across various fields [1–3]. Since the emergence of models like ChatGPT [4–6], LLMs have shown tremendous potential in healthcare, attracting widespread attention from both academic and industrial sectors [7, 8]. Traditional Chinese Medicine (TCM), with its millennia-long history, offers a unique theoretical framework, diagnostic methods, and rich experiential knowledge. While TCM has made important contributions to human healthcare, it also faces challenges in knowledge inheritance, standardization, and modernization. How to leverage LLMs and other AI technologies to facilitate the digital preservation, intelligent dissemination, and innovative development of TCM knowledge has become a key scientific question in the modernization process of TCM [9–11].

With the rapid iteration of LLMs technology and deeper applications in vertical domains, research on LLMs in TCM has been increasing annually, with numerous professional LLMs emerging for TCM clinical practice [12–18]. These models demonstrate value in knowledge consultation, diagnostic assistance, and prescription recommendations by learning from multiple data sources including TCM classics, modern textbooks, clinical data, and professional knowledge graphs. However, current research remains fragmented and highly heterogeneous, lacking review and comprehensive analysis. Furthermore, there is no consensus on key aspects such as model tuning techniques, data construction strategies, evaluation method design, and application scenario positioning, which constrains the collaborative development and clinical transformation of TCM-oriented LLMs. Simultaneously, TCM, as a medical system that integrates holistic, dialectical, and experiential characteristics, with its holistic concepts and personalized "syndrome differentiation and treatment" approach, differs from the knowledge representation methods of modern LLMs based on statistical models. Directly applying LLMs research paradigms from Western medicine faces numerous adaptation challenges.

Although several recent reviews have examined LLMs applications in Traditional Chinese Medicine [9, 19–25], existing literature primarily focuses on describing application scenarios and assessing model performance in specific tasks. However, these reviews lack systematic evaluation of the technical foundation underlying TCM LLMs development—specifically, the selection rationale for model tuning techniques, data construction strategies, training processes, and evaluation framework design. Furthermore, there is insufficient analysis of how different technical choices affect model performance in TCM-specific tasks, nor comprehensive examination of the challenges inherent to adapting LLM architectures for TCM's unique epistemological framework.

Based on these considerations, this study adopts a scoping review methodology to systematically examine the current status of LLMs tuning and applications in TCM clinical practice. We analyze the applicability of different tuning techniques, data construction strategies, and evaluation methods in the TCM field. We also explore the value and limitations of TCM-oriented LLMs in knowledge consultation and diagnostic assistance, and summarize existing evaluation benchmarks for TCM-focused LLMs, while proposing future research directions and development recommendations.

## Methods

This research employs a scoping review methodology, following the standard reporting checklist of PRISMA Extension for Scoping Reviews (PRISMA-ScR) [26]. We comprehensively evaluate the current applications, challenges, and future directions of LLMs in TCM clinical practice.

### Search strategy

We conducted a systematic computer search for studies related to LLMs applications in TCM. Databases searched included PubMed, Web of Science, IEEE Xplore, ACM Digital Library, arXiv, CNKI, and Wanfang Database. The search strategy consisted of two groups of terms. Using English search as an example: LLMs-related terms ("large language model*", "LLM*", "Generative Pre-trained Transformer", "generative AI", "Fine-tuning", "Reinforcement Learning", "Direct Preference Optimization", "Supervised Learning", "Proximal Policy Optimization", "Low-Rank Adaptation", "Attention Mechanism", "Pre-training") and TCM-related terms ("Traditional Chinese Medicine", "TCM", "Chinese herbal medicine", "herbal formula*", "acupuncture", "moxibustion", "cupping", "Gua Sha", "scraping", "tuina", "massage", "external therapy", "manipulation"). The search period extended from database inception to May 2025. Complete search strategies for all database are provided in Supplementary Table S1.

### Inclusion and exclusion criteria

Inclusion criteria: (1) Original research, systematic reviews, meta-analyses, and similar literature; (2) Studies that adapted LLMs through various tuning techniques (including fine-tuning, retrieval-augmented generation, prompt engineering, or continued pre-training) for application in TCM clinical practice or constructed TCM LLMs evaluation benchmarks; (3) Literature in Chinese or English. Exclusion criteria: (1) Non-academic articles (news reports, blogs, editorials, etc.); (2) Literature that merely mentioned or used LLMs without applying model optimization techniques; (3) Literature with inaccessible full text; (4) Conference abstracts (unless providing detailed methods and results); (5) Duplicate publications.

### Literature screening process

EndNote X9 software was used to process initial search results and remove duplicates. Two independent researchers with backgrounds in TCM and artificial intelligence screened the literature according to the predetermined inclusion and exclusion criteria, primarily based on titles and abstracts. For literature potentially meeting the inclusion criteria, researchers conducted full-text readings to make final decisions. Disagreements during the screening process were resolved through discussion with a third senior researcher. We recorded in detail the number of papers identified, screened, eligible, and finally included.

### Data extraction

Data extraction was independently performed by two researchers using a pre-designed standardized form to collect relevant information. Extracted data included basic paper information (title, first author, publication year, country/region, application scenario, research objectives, main results), model and data information (base model and parameters, LLMs techniques, data characteristics), model evaluation (evaluation methods and metrics, comparison models, evaluation data), and research limitations and future prospects. For TCM evaluation benchmark studies, additional information was extracted on benchmark construction methods, test dimensions, number of questions, and test results. The extracted data underwent cross-verification to ensure accuracy and consistency. Disagreements were resolved through discussion between the two researchers, with consultation of third-party experts or original authors when necessary to ensure research data accuracy and completeness.

### Statistical methods

This study employed descriptive statistical analysis methods. We performed statistical analysis on the basic information and model data of included studies, presenting results through frequency statistics, composition ratios, and textual and visual presentations.

## Results

### Literature screening results

**Fig. 1:**
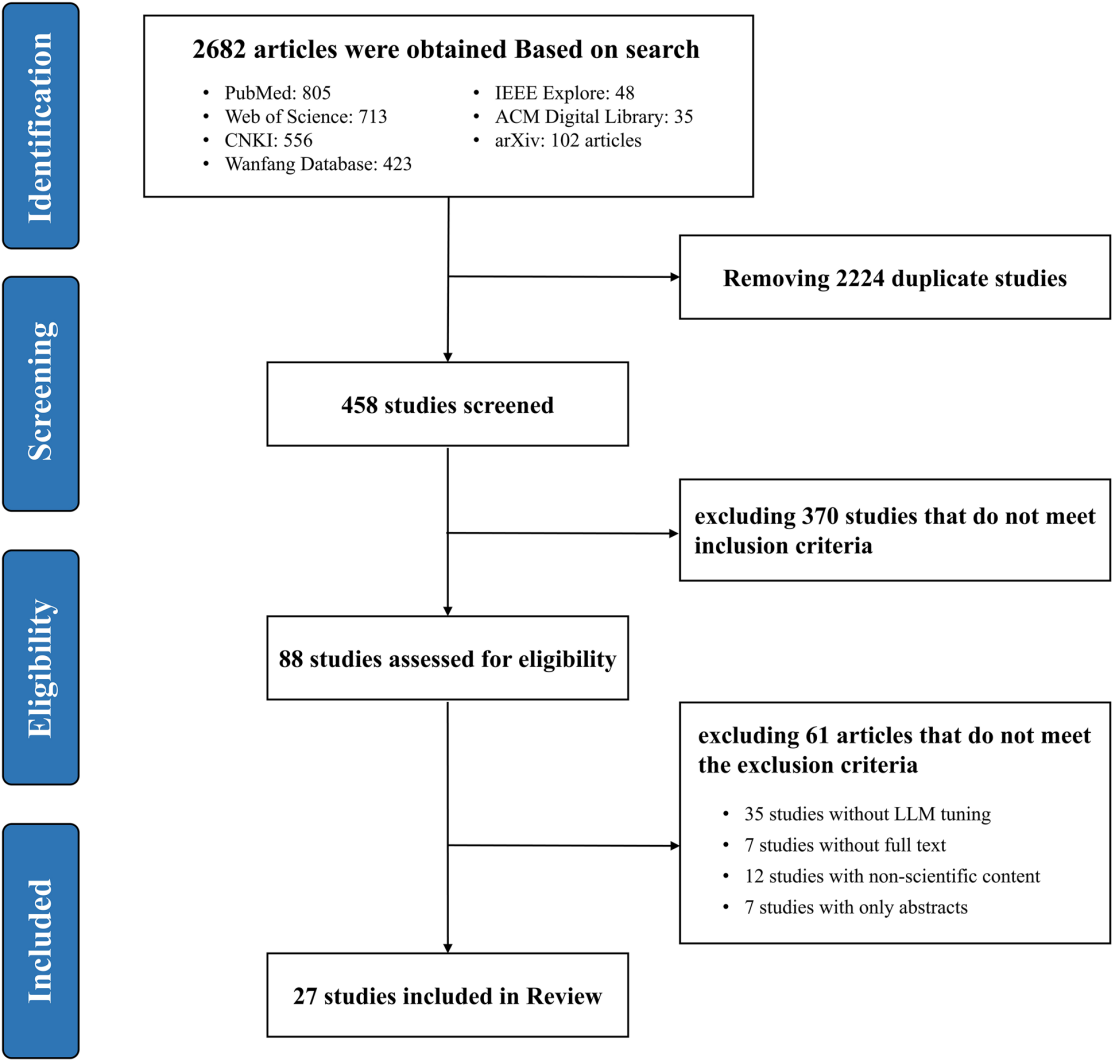
Literature screening flow diagram based on the PRISMA statement

Through systematic searches across databases, we retrieved a total of 2682 research articles related to LLMs in TCM clinical practice. Using Endnote X9 for duplicate checking, combined with manual review, we removed 2224 duplicate articles, leaving 458 articles. Further initial screening of titles and abstracts eliminated 370 articles that did not meet the inclusion criteria. We then downloaded and reviewed the full text of the remaining 88 articles, excluding 35 that did not involve LLMs tuning, 7 with inaccessible full texts, 12 non-academic articles, and 7 conference abstracts. A final total of 27 articles were included, with the literature screening process shown in Fig. 1. Detailed information on model characteristics, tuning techniques, data sources, evaluation methods, and application domains for all included studies is presented in Supplementary Tables S2-S4.

### Basic characteristics of included studies

Among the 27 included articles, 21 were in English and 6 in Chinese. (i) Publication year: 4 articles (14.8%) were published in 2023, 20 articles (74.1%) in 2024, and 3 articles (11.1%) in 2025. (ii) Article type: All 27 articles were research papers, including 5 computer conference papers, 12 journal articles, and 10 preprints. (iii) Publication region: 26 studies were from China, and 1 study was from Malaysia.

### LLM application domains

**Fig. 2:**
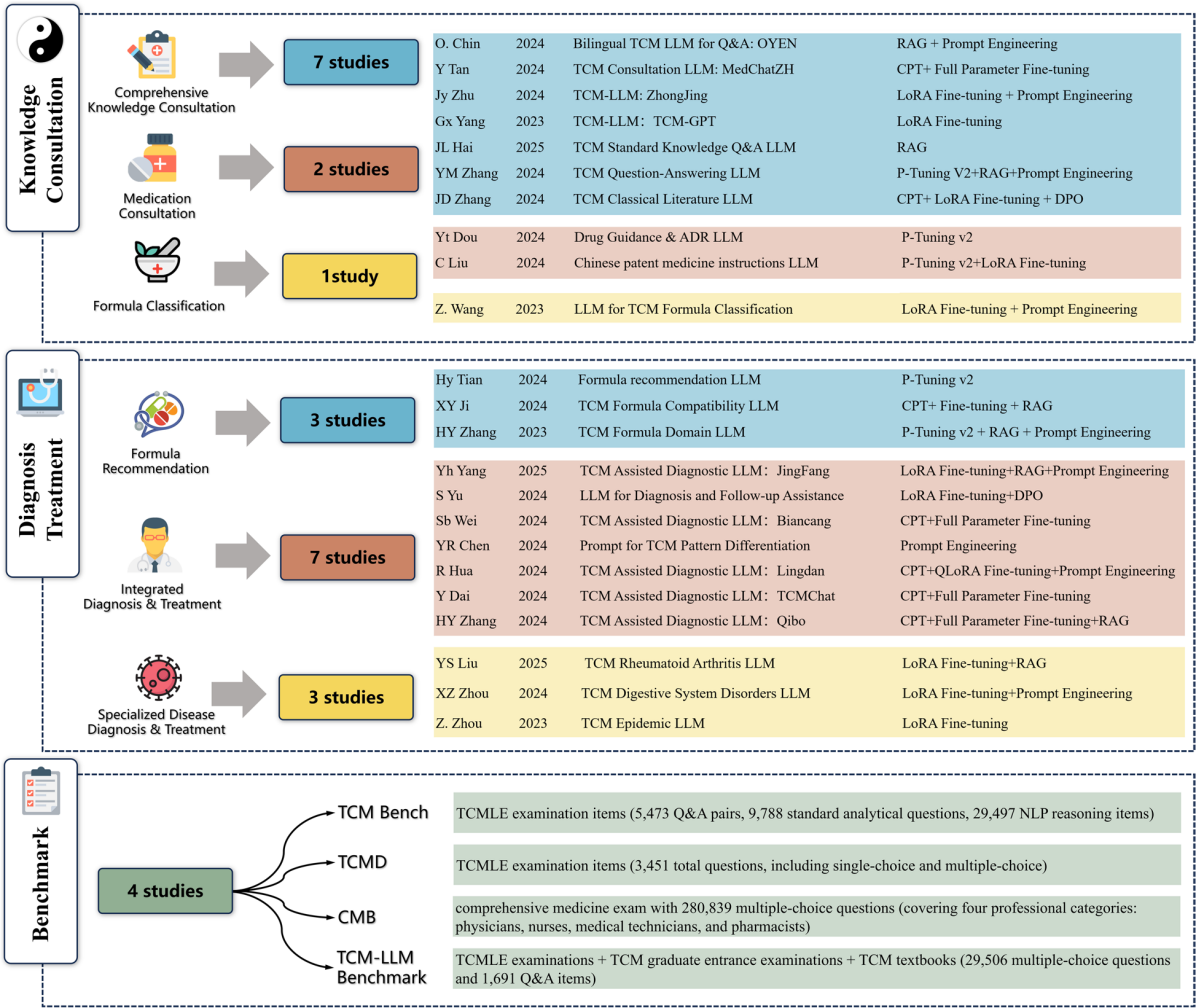
TCM Large Language Model applications. Shows knowledge consultation, diagnostic assistance, and testing benchmarks with specific research in each subcategory; each study lists authors, publish years, core content, and fine-tuning techniques used. CMB: Comprehensive Medical Benchmark, CPT: Continued Pre-Training, DPO: Direct Preference Optimization, LLM: Large Language Model, LoRA: Low-Rank Adaptation, NLP: Natural Language Processing, QLoRA: Quantized Low-Rank Adaptation, RAG: Retrieval-Augmented Generation, TCM: Traditional Chinese Medicine, TCMD: Traditional Chinese Medicine QA Dataset. TCMLE: Traditional Chinese Medicine Licensing Exam

Based on the research topics and application domains of the included literature, we classified them into two major categories: TCM knowledge consultation and diagnosis & Treatment assistance. Knowledge consultation was further divided into: (i) comprehensive knowledge consultation (7 studies) [12, 13, 27–31], (ii) formula classification (1 study) [32], and (iii) medication consultation (2 studies) [15, 17]. Diagnosis & Treatment assistance was subdivided into: (i) formula recommendation (3 studies) [33–35], (ii) integrated diagnosis & treatment (7 studies) [14, 16, 36–40], and (iii) specialized disease diagnosis & treatment (3 studies) [41–43]. Additionally, considering the diversification, complexity, and lack of standardization in the evaluation and testing of LLMs in vertical domains, we also included 4 TCM LLM benchmark studies [44–47] (Fig. 2).

### TCM large language model tuning techniques

The effectiveness of LLMs in the TCM field largely depends on the selection and implementation of model tuning technique [48, 49]. This section systematically analyzes the tuning techniques employed in 23 studies (excluding 4 TCM LLM benchmark testing studies) to explore the application and effects of different tuning methods.

#### Introduction to LLM tuning techniques

**Fig. 3:**
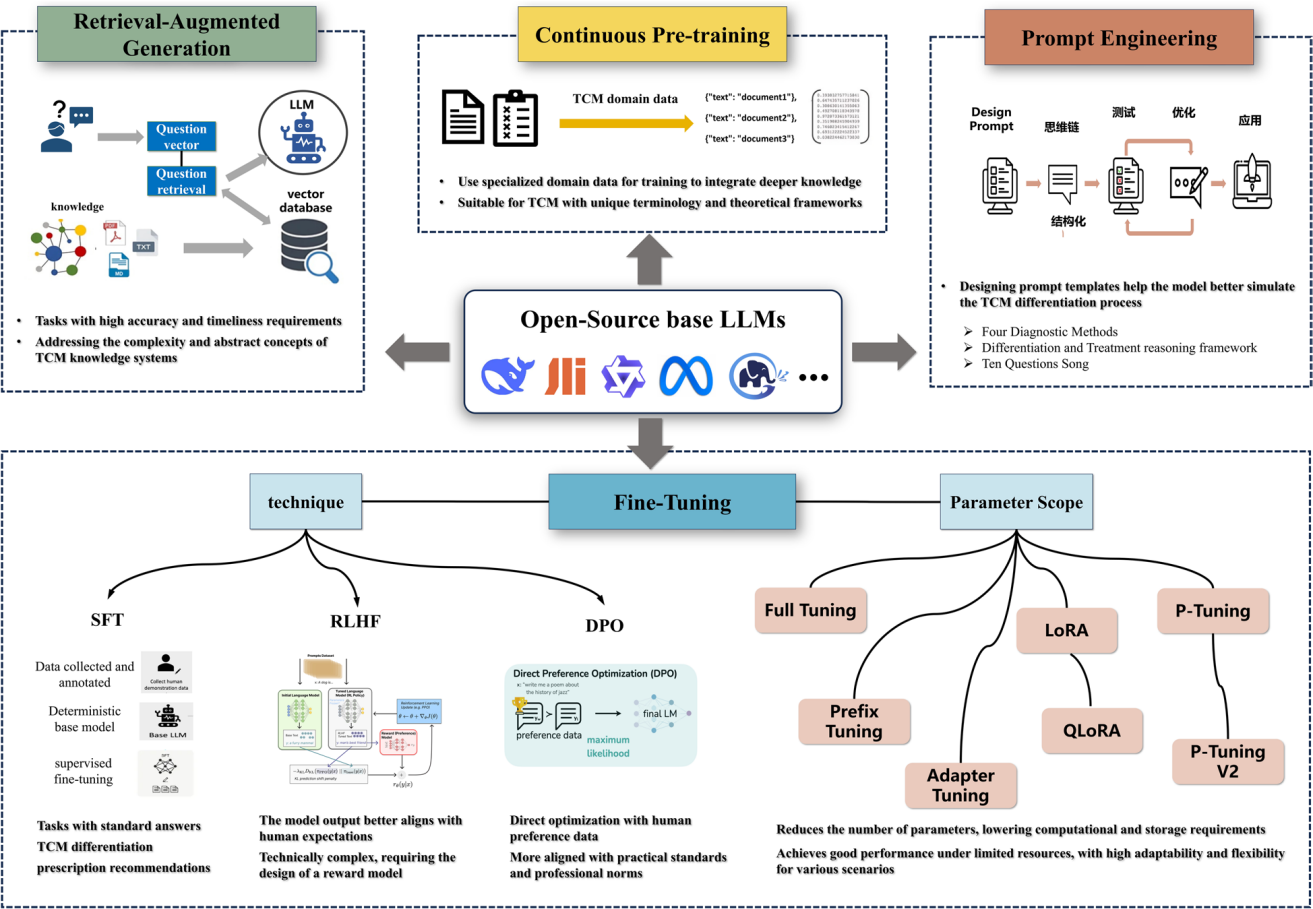
Common tuning Techniques and Their Advantages in the TCM Field. DPO: Direct Preference Optimization, LLMs: Large Language Models, LoRA: Low-Rank Adaptation, QLoRA: Quantized Low-Rank Adaptation, RLHF: Reinforcement Learning from Human Feedback, SFT: Supervised Fine-Tuning, TCM: Traditional Chinese Medicine

Based on technical characteristics and operational mechanisms, LLM tuning techniques commonly used in the TCM field primarily fall into four categories: continued pre-training [50–52], fine-tuning [53–57], retrieval-augmented generation [58], and prompt engineering [59]. Continued pre-training focuses on domain adaptation at the knowledge level, enabling models to acquire richer TCM professional knowledge. Fine-tuning techniques optimize capabilities for specific tasks, including supervised fine-tuning [48, 53], reinforcement learning [54–56], direct preference optimization [57], and parameter update scopes such as full-parameter fine-tuning, LoRA [60], and QLoRA [61]. Retrieval-augmented generation techniques address the limitations of model parameter knowledge by introducing external knowledge sources. Prompt engineering requires no parameter changes, guiding models to generate outputs conforming to TCM diagnostic standards through carefully designed instructions. In practical TCM applications, researchers often adopt multiple techniques in combination, such as continued pre-training followed by LoRA fine-tuning, or combining RAG with prompt engineering to enhance model performance for rare conditions. Table S5 and Fig. 3 summarize the core characteristics, advantages, and application value of each type of tuning technique in the TCM field.

#### Application of tuning techniques in TCM LLMs

(1) Single and Combined Techniques: Only 3 studies (13.0%) employed a single tuning technique, while the remaining 20 studies (87.0%) utilized combinations of two or more techniques. The most common combination patterns were "parameter-efficient tuning + prompt engineering" (30.4%) and "continued pre-training + parameter-efficient tuning" (26.1%). (2) Frequency of Different Techniques: LoRA fine-tuning was most widely applied, accounting for 65.2% (15/23); followed by prompt engineering (47.8%, 11/23), continued pre-training (43.5%, 10/23), RAG technology (39.1%, 9/23), full-parameter fine-tuning (30.4%, 7/23), P-Tuning v2 (26.1%, 6/23), and DPO (8.7%, 2/23). (3) Relationship Between Technique Selection and Model Scale: For models with 7B parameters and below, both parameter-efficient tuning (LoRA, P-Tuning v2) and full-parameter tuning were applied; for models with 13B parameters and above, parameter-efficient tuning predominated (93.3%), with only a few studies using full-parameter tuning (6.7%).

### TCM large language model training data analysis

#### Training data types and sources

Training data in the included studies primarily came from seven major sources: clinical case data (73.9%, 17 studies), TCM classics and textbooks (65.2%, 15 studies), TCM standards and pharmacopoeias (43.5%, 10 studies), public healthcare datasets (39.1%, 9 studies), web-crawled data (30.4%, 7 studies), professional examination question banks (26.1%, 6 studies), and professional knowledge graphs (21.7%, 5 studies). The combination of these diverse data sources aimed to build comprehensive training corpora that fully reflect both TCM theoretical systems and clinical practices.

#### Data differences across application domains

Training data composition varied significantly across different TCM application domains. Knowledge consultation applications primarily relied on classical literature, textbooks, and professional Q&A datasets (e.g., ZhongJing model used 1.12 million Q&A pairs, Huang-Di model incorporated 500,000 ancient text dialogues). Formula classification and prescription recommendation applications focused on structured formula data, pharmacopoeias, and clinical medication records, emphasizing precise information on composition, indications, and dosages (e.g., Wang et al.'s formula classification study used 2617 formula entries, TCM-FTP built a dataset with 18,953 digestive disease cases). Diagnostic assistance applications extensively utilized clinical case data and treatment records, prioritizing authentic clinical experience (e.g., BianCang model used approximately 228 M tokens of tuning data, JingFang model incorporated 43,000 clinical cases). Medication guidance applications combined pharmaceutical data with clinical usage records, emphasizing medication safety and accuracy (e.g., ShennongMGS system integrated 22,327 clinical cases and 13,020 medical knowledge Q&A entries). This targeted data selection significantly influenced model performance in their respective application scenarios.

### Large language model evaluation metrics

#### Introduction to common evaluation metrics

Evaluation metrics for TCM-oriented LLMs fell into three main categories. First, accuracy-related metrics were widely used, with accuracy [62] being the most common (17 studies, 63.0%), particularly suitable for classification tasks like TCM licensing examinations and syndrome differentiation. Precision and recall [63] (10 studies, 37.0%) were primarily employed for TCM entity recognition and formula compatibility tasks, while F1 score [64] (9 studies, 33.3%) provided a comprehensive performance assessment by averaging precision and recall.

Natural language generation metrics constituted the second category, including BLEU [65] (9 studies, 33.3%), which evaluates generation quality through n-gram overlap between model outputs and reference texts, particularly for knowledge question-answering and prescription generation tasks. ROUGE [66] (10 studies, 37.0%) assessed vocabulary and sequence overlap in generated texts, while less frequently used metrics included METEOR [67] (2 studies), which considers synonym matching, and BERTScore [68] (3 studies), which captures semantic similarity through contextual embeddings.

**Fig. 4:**
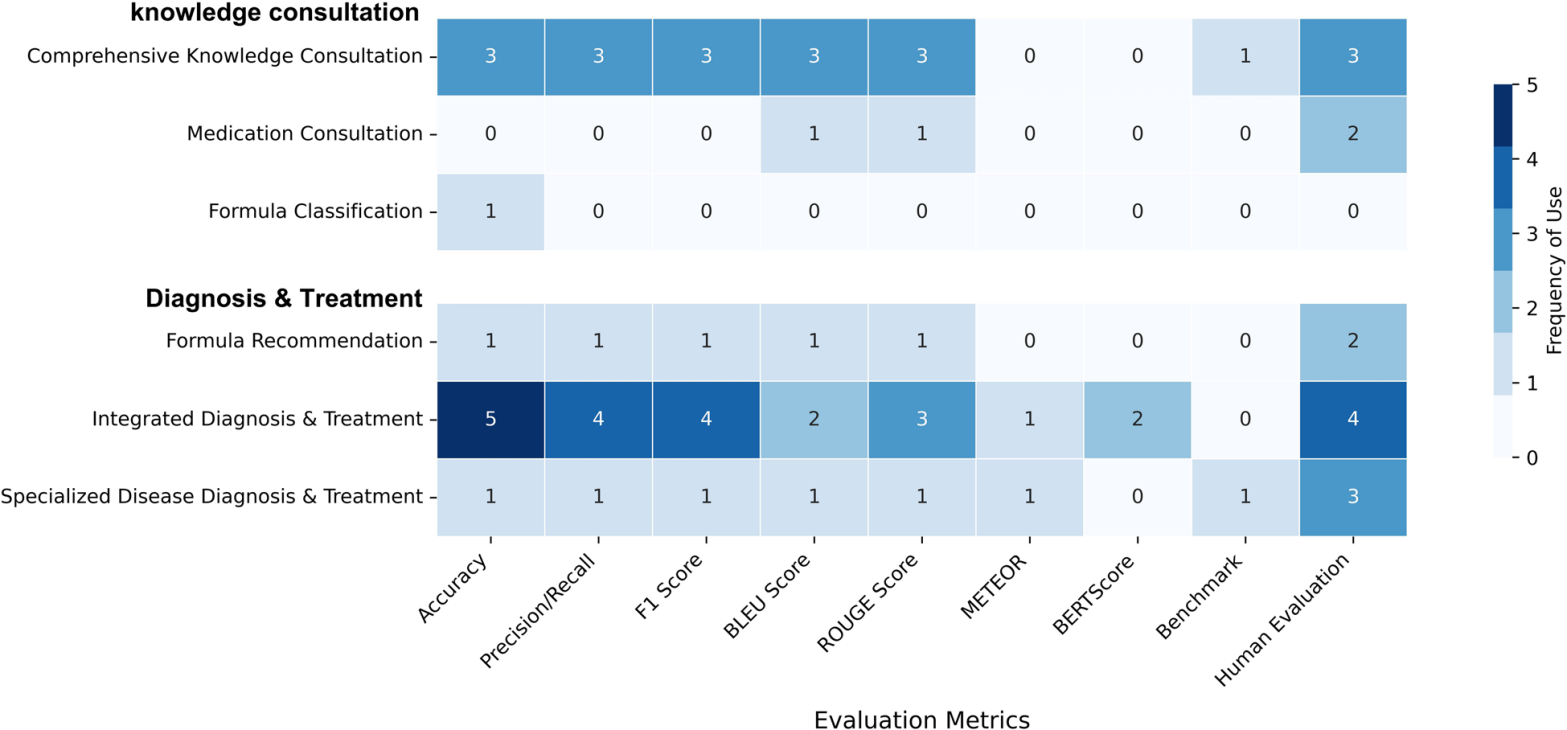
Heatmap of Evaluation Metrics Usage Across Different TCM LLM Application Domains. BERT: Bidirectional Encoder Representations from Transformers, BLEU: Bilingual Evaluation Understudy, ROUGE: Recall-Oriented Understudy for Gisting Evaluation, METEOR: Metric for Evaluation of Translation with Explicit Ordering

Due to the unique characteristics of domain-specific generative LLMs, human evaluation emerged as the most important assessment approach [69], employed in 21 studies (77.8%). Human evaluation typically involved expert scoring by TCM specialists across multiple dimensions (professionalism, accuracy, reasonableness, completeness); consistency assessment to verify evaluation reliability; Turing tests comparing model-generated content with human expert outputs; and multi-dimensional assessment evaluating aspects such as fluency, relevance, completeness, and medical expertise. The distribution of evaluation metrics across different TCM LLM application domains is shown in Fig. 4.

#### TCM domain-specific evaluation methods

As TCM-oriented LLM applications have developed, generic evaluation methods have struggled to fully reflect model performance in TCM-specific scenarios. Consequently, several studies have established specialized TCM domain evaluation methods and test sets. These methods better align with TCM knowledge characteristics and clinical practices, providing more precise evaluation tools for TCM-oriented LLMs. These include the four evaluation benchmarks mentioned earlier (TCMBench, TCMD, CMB, TCM LLM evaluation benchmark dataset), detailed in Table 2, as well as other evaluation methods specifically constructed in conjunction with model applications: TCM Eval and TCM Score.

### Performance limitations and common challenges in TCM-oriented LLMs

#### Suboptimal performance on standardized benchmarks

The four benchmark evaluation studies revealed concerning performance gaps across all tested models. Yue et al.'s TCMBench evaluation found that all LLMs failed to meet satisfactory thresholds, with GPT-4 achieving only 59.86% accuracy [43]. Cao et al.'s evaluation using 31,197 exam questions demonstrated that all models achieved less than 60% accuracy, with general LLMs slightly outperforming Chinese medical LLMs [46]. Yu et al.'s TCMD benchmark showed general-purpose LLMs unexpectedly outperforming TCM-specific LLMs [44], challenging assumptions about domain specialization effectiveness and suggesting potential overfitting or inadequate training data quality. Wang et al.'s CMB showed significant accuracy variations between knowledge domains [45]. These findings indicate current models have not achieved the knowledge depth and reasoning capabilities required for reliable clinical deployment.

#### Complex reasoning and clinical decision-making deficits

Studies documented systematic difficulties in executing complex TCM reasoning. Multiple studies revealed struggles with syndrome differentiation, with Wei et al.'s BianCang achieving 78.90% accuracy [38], Yan et al.'s JingFang reaching F1-score of 0.8186 [35], and Zhang et al.'s Qibo achieving Rouge-L scores of 0.55–0.72 [36]—indicating 20–30% error rates in this cornerstone diagnostic process. Models struggle to master the complete chain from "four examinations integration" to "syndrome differentiation and treatment," requiring simultaneous balancing of syndrome identification accuracy, treatment rationality, prescription standardization, and individualized adjustments. Beyond syndrome differentiation, models demonstrated limited holistic thinking, with studies noting that models fragment TCM knowledge rather than capturing systemic connections [15, 17].

#### Information accuracy and hallucination concerns

Evidence of hallucination—generating plausible but factually incorrect information—emerged consistently as a critical barrier. The prevalence of retrieval-augmented generation (RAG) implementation in 9 of 23 studies (39.1%) reflects explicit recognition of this challenge. Zhang et al. specifically developed RAG-based systems to "reduce hallucinations" [30]. Hai et al. positioned RAG as essential for ensuring response accuracy [29]. Hallucination is a systematic challenge inherent to applying LLMs in TCM rather than an isolated issue. This challenge is compounded by TCM's vast pharmacopoeia, intricate formula compatibility rules, and syndrome-specific treatment principles.

## Discussion

### Main findings

**Fig. 5:**
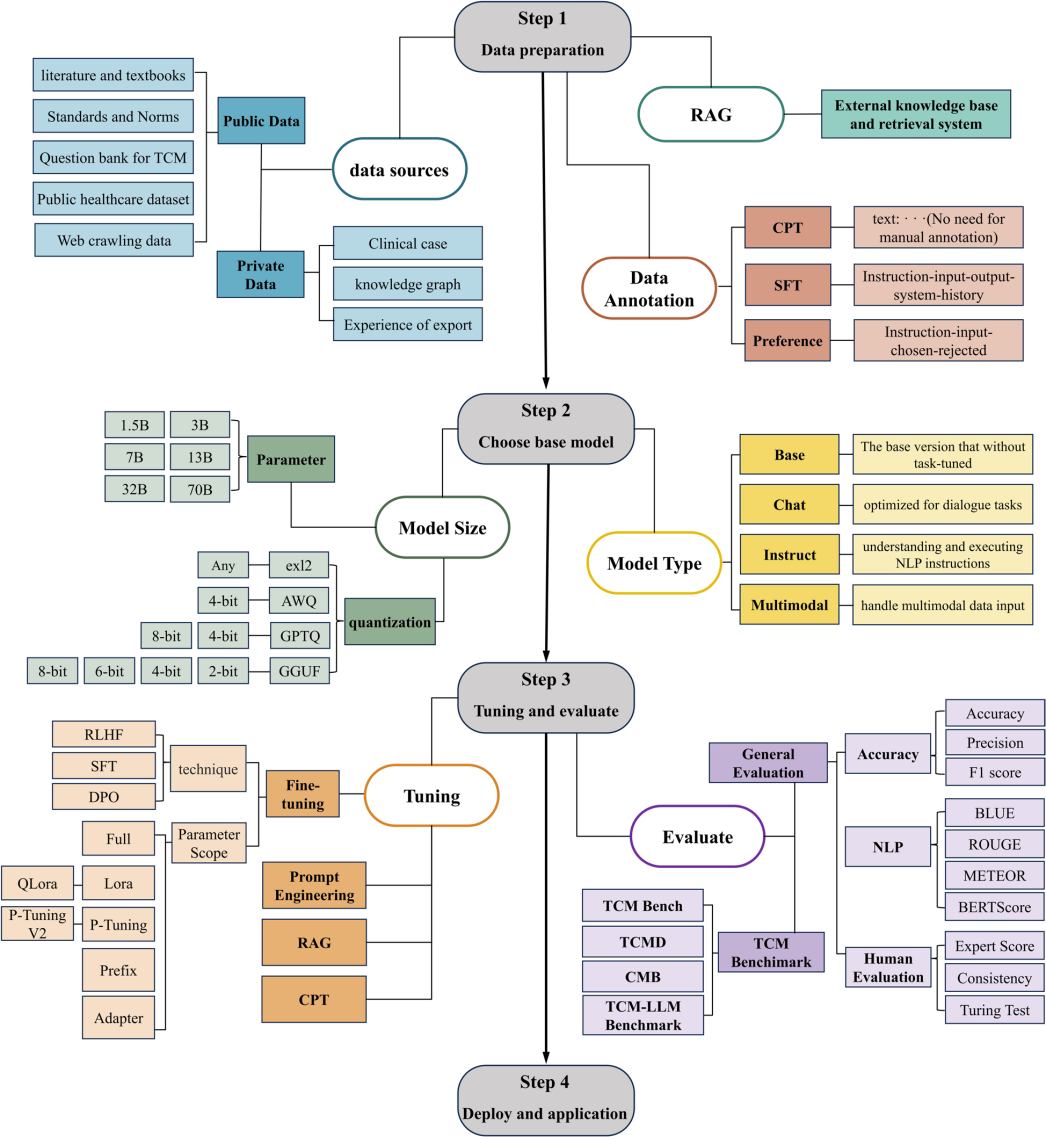
Flowchart of TCM Large Language Models Fine-tuning and Application Process. BERT: Bidirectional Encoder Representations from Transformers, BLEU: Bilingual Evaluation Understudy, CMB: Comprehensive Medical Benchmark, CPT: Continued Pre-Training, DPO: Direct Preference Optimization, LoRA: Low-Rank Adaptation, METEOR: Metric for Evaluation of Translation with Explicit Ordering, NLP: Natural Language Processing, QLoRA: Quantized Low-Rank Adaptation, RAG: Retrieval-Augmented Generation, RLHF: Reinforcement Learning from Human Feedback, ROUGE: Recall-Oriented Understudy for Gisting Evaluation, SFT: Supervised Fine-Tuning, TCM: Traditional Chinese Medicine, TCMD: Traditional Chinese Medicine QA Dataset

Through systematic review of 27 studies on LLM tuning and applications in TCM, this research reveals key achievements, technical approaches, existing challenges, and future directions. And we systematically summarized the workflow for LLM tuning and application in the TCM field, as shown in Fig. 5. TCM LLM tuning primarily focuses on knowledge consultation and diagnostic assistance, with technical approaches showing a trend toward multi-method combinations, predominantly parameter-efficient fine-tuning (especially LoRA). Data construction balances theoretical knowledge (TCM classics) with practical experience (clinical cases), though multimodal data remains severely insufficient. Evaluation methods are comprehensive and multidimensional, with specialized TCM assessment benchmarks (such as TCMBench and TCMD) gradually being established. Current TCM-oriented LLMs demonstrate excellent performance in integrating heterogeneous knowledge sources, simulating basic syndrome differentiation reasoning, and cross-language knowledge conversion, opening new pathways for digital preservation and intelligent transmission of TCM knowledge systems. The widespread application of various LLM tuning techniques indicates that TCM LLM research is developing toward balancing resource efficiency with performance, progressively overcoming computational resource constraints. However, a critical gap emerges in domain-specific applications: only 3 of 27 studies (11.1%) targeted specific disease domains (rheumatoid arthritis, epidemic diseases, digestive system diseases), while the majority focus on general TCM knowledge processing. This lack of subspecialty differentiation reflects both TCM's holistic medical paradigm and the current nascent stage of the field, where high-quality domain-specific clinical data remain scarce. Nevertheless, research is progressively deepening from general knowledge services to specific clinical scenarios, reflecting increased technical maturity and a promising trajectory toward application specificity.

### Key patterns and their implications in TCM LLMs

This systematic analysis of 27 studies reveals three critical patterns shaping TCM LLM development. First, LoRA dominates parameter-efficient methods (65.2%), with multi-technique combinations prevalent (87%), reflecting resource constraints rather than evidence-based selection [70]. Second, severe multimodal data scarcity exists—studies rely predominantly on text despite TCM's requirement for integrated diagnostic inputs including tongue images, pulse recordings, and facial features [71]. Third, evaluation methods emphasize accuracy metrics (63% of studies) and knowledge recall, failing to assess holistic reasoning and syndrome differentiation capabilities distinguishing expert practitioners. These patterns reflect systematic challenges in adapting general-purpose LLMs to TCM's unique knowledge structure and clinical paradigm.

### General LLM limitations in TCM applications

Three fundamental limitations constrain TCM LLM development. First, computational constraints drive adoption of parameter-efficient methods without validating their suitability for TCM tasks. Comparative research evaluating full fine-tuning, LoRA, QLoRA, and DPO across TCM applications (knowledge Q&A, syndrome differentiation, prescription generation) remains absent [70–72]. Hyperparameter optimization studies are similarly lacking, leaving unclear whether LoRA dominance reflects genuine superiority or computational necessity.

Second, data ecosystem deficiencies create integration barriers. Severe standardization gaps—heterogeneous formats, annotation methods, and terminology systems—prevent collaborative development. Multimodal data scarcity is particularly critical: as described in the training data analysis, text-based sources dominance (clinical cases 73.9%, TCM classics 65.2%) despite TCM's "four diagnostic methods" requiring visual, auditory, olfactory, tactile, and linguistic inputs. This text-only focus fundamentally limits authentic TCM diagnostic simulation. Data accessibility barriers compound these issues—most studies do not report acquisition paths or open-source status, causing redundant investment.

Third, evaluation methodology limitations persist despite specialized TCM benchmarks (TCMBench, TCMD, TCM-3CEval, as discussed in the evaluation metrics section). Current frameworks prioritize laboratory performance over clinical complexity, failing to verify mastery of complete reasoning chains from "four examinations integration" to "syndrome differentiation and treatment." Human evaluation lacks standardization: variations in evaluator composition, professional backgrounds, scoring dimensions, and assessment protocols, combined with absent inter-rater reliability reporting and lack of blinded designs, reduce result credibility and cross-study comparability. These limitations—spanning computational resources, data quality, and evaluation rigor—intensify when intersecting with TCM's unique epistemological paradigm.

### TCM-specific knowledge and reasoning challenges

TCM presents distinctive challenges beyond general LLM limitations. TCM's core concepts—"holistic view" and "syndrome differentiation and treatment"—fundamentally differ from statistical pattern-matching in modern LLM architectures. Models demonstrate proficiency in basic tasks but significant deficiencies in complex TCM reasoning chains, systemic multi-dimensional thinking, and individualized diagnosis [72]. Quantitative evidence demonstrates this gap: TCM-3CEval benchmarking shows 51.23% accuracy on memorization tasks (herb properties) versus 32.18% on syndrome-based clinical reasoning, revealing how TCM's emphasis on contextual symptom relationships—rather than isolated symptom-disease correlations—creates distinctive cognitive challenges. This non-linear, systemic knowledge system resists expression through current transformer architectures, causing models to fragment TCM knowledge rather than capture inherent theoretical connections [73].

TCM diagnosis requires pattern recognition across seemingly unrelated symptoms, guided by Yin-Yang balance, Five Elements correspondence, and Zang-Fu organ system [74]. Models lacking exposure to these frameworks struggle with syndrome differentiation requiring holistic pattern recognition over linear algorithms [75]. Syndrome differentiation complexity—"multiple syndromes coexisting," "same disease different treatments," "different diseases same treatment"—creates evaluation dilemmas where establishing single correct answers proves problematic. Current transformer attention mechanisms inadequately model long-sequence dependencies and nonlinear causal relationships in TCM diagnostic chains spanning hundreds of inferential steps [40]. Moreover, TCM's experiential nature, refined through centuries of clinical practice and master-apprentice transmission, resists formalization. The training data composition reported above (clinical cases 73.9%, classics 65.2%) captures explicit knowledge but struggles with implicit diagnostic intuitions, subtle pattern recognition, and context-dependent treatment adjustments characterizing expert practice. Existing tuning techniques inadequately represent TCM's holistic thinking, lacking frameworks reflecting diagnostic workflows integrating "four examinations" into coherent syndrome-pattern identification and individualized treatment formulation.

### Recommendations for future research

1. Establish systematic tuning methodology evaluation frameworks. Conduct rigorous comparative studies of full fine-tuning, LoRA, QLoRA, and DPO across diverse TCM tasks (knowledge Q&A, syndrome differentiation, prescription generation), with standardized hyperparameter optimization protocols and computational efficiency benchmarking to replace empirical selection with evidence-based methodology.
2. Build comprehensive multimodal TCM data ecosystems. Systematically collect integrated diagnostic data (tongue images, pulse recordings, facial features, auditory cues) aligned with TCM's "four diagnostic methods," establish unified annotation standards and open-access platforms, and develop incentive mechanisms promoting data sharing to address current text-only limitations.
3. Develop TCM-aligned evaluation frameworks. Design multi-dimensional assessment protocols testing holistic reasoning capabilities—including syndrome differentiation logic, formula-syndrome correspondence, and pattern recognition across seemingly unrelated symptoms—rather than isolated knowledge recall. Standardize human evaluation procedures with explicit inter-rater reliability reporting and blinded assessment designs.
4. Explore TCM-specific architectural innovations. Investigate integration of TCM theoretical constructs (Yin-Yang balance, Five Elements correspondence, Zang-Fu systems) into model architectures through specialized attention mechanisms, knowledge graph-enhanced reasoning modules, and temporal frameworks modeling syndrome evolution and treatment adjustment processes.
5. Strengthen expert knowledge integration and clinical translation. Transform implicit TCM diagnostic intuitions into structured training signals through expert knowledge distillation and clinical experience annotation. Conduct prospective clinical validation studies with clear ethical frameworks, establishing human-AI collaboration models that preserve TCM's individualized, holistic diagnostic paradigm while meeting modern regulatory requirements.

### Study limitations

This scoping review has the following limitations: First, approximately 37% of included studies are preprints that have not undergone peer review, reflecting the nascent stage and rapid development of TCM LLMs research between 2023–2025. Second, included studies show high heterogeneity, with significant differences in evaluation metrics, data sources, and research designs, limiting quantitative comparative analysis. Additionally, our search was limited to English and Chinese literature, which may exclude relevant studies published in other languages. Finally, this review primarily focuses on application and technical analysis, with relatively limited discussion of ethical, legal, and social impacts of TCM LLMs.

## Supplementary Information

Supplementary Material 1

## Data Availability

All data used in this review are fully available in the public domain.

## Abbreviations

AI: Artificial Intelligence
BLEU: Bilingual evaluation understudy
CMB: Comprehensive medical benchmark
CPT: Continued pre-training
DPO: Direct preference optimization
ICLR: International conference on learning representations
LLMs: Large language models
LoRA: Low-Rank Adaptation
METEOR: Metric for Evaluation of Translation with Explicit Ordering
NLP: Natural Language Processing
PE: Prompt Engineering
QLoRA: Quantized Low-Rank Adaptation
RAG: Retrieval-augmented generation
RLHF: Reinforcement learning from human feedback
ROUGE: Recall-oriented understudy for gisting evaluation
SFT: Supervised fine-tuning
TCM: Traditional Chinese Medicine
TCMD: Traditional Chinese Medicine QA Dataset
TCMEval: Traditional Chinese Medicine evaluation framework
TCMLE: Traditional Chinese Medicine licensing exam

## References

[1] Vaswani A, Shazeer N, Parmar N, Uszkoreit J, Jones L, Gomez AN, et al. Attention is all you need. Proceedings of the 31st International Conference on Neural Information Processing Systems; Long Beach, California, USA: Curran Associates Inc. 2017.

[2] Devlin J, Chang M-W, Lee K, Toutanova K, editors. BERT: Pre-training of Deep Bidirectional Transformers for Language Understanding2019 June; Minneapolis, Minnesota: Association for Computational Linguistics.

[3] Radford A, Narasimhan K. Improving Language Understanding by Generative Pre-Training. 2018.

[4] Brown TB, Mann B, Ryder N, Subbiah M, Amodei D. Language models are few-shot learners. Adv Neural Inf Process Syst. 2020;33:1877–901.

[5] Touvron H, Lavril T, Izacard G, Martinet X, Lachaux M-A, Lacroix T, et al. LLaMA: Open and Efficient Foundation Language Models. 2023;abs/2302.13971.

[6] Radford A, Wu J, Child R, Luan D, Amodei D, Sutskever I. Language models are unsupervised multitask learners. OpenAI blog. 2019;1(8):9.

[7] Bedi S, Liu Y, Orr-Ewing L, Dash D, Koyejo S, Callahan A, et al. Testing and evaluation of health care applications of large language models: a systematic review. JAMA. 2025;333(4):319–28. 39405325 10.1001/jama.2024.21700 PMC11480901

[8] Wang L, Wan Z, Ni C, Song Q, Li Y, Clayton E, et al. Applications and concerns of ChatGPT and other conversational large language models in health care: systematic review. J Med Internet Res. 2024;26:e22769. 39509695 10.2196/22769 PMC11582494

[9] Ren Y, Luo X, Wang Y, Li H, Zhang H, Li Z, et al. Large language models in traditional Chinese medicine: a scoping review. J Evid Based Med. 2025;18(1):e12658. 39651543 10.1111/jebm.12658

[10] Zhang S, Wang W, Pi X, He Z, Liu H. Advances in the application of traditional Chinese medicine using artificial intelligence: a review. Am J Chin Med. 2023;51(5):1067–83. 37417927 10.1142/S0192415X23500490

[11] Li N, Yu J, Mao X, Zhao Y, Huang L. The Research and Development Thinking on the Status of Artificial Intelligence in Traditional Chinese Medicine. Evid Based Complement Alternat Med. 2022;2022:7644524. 35547656 10.1155/2022/7644524 PMC9085309

[12] Zhu J, Gong Q, Zhou C, Luan H. ZhongJing: A Locally Deployed Large Language Model for Traditional Chinese Medicine and Corresponding Evaluation Methodology: A Large Language Model for data fine-tuning in the field of Traditional Chinese Medicine, and a new evaluation method called TCMEval are proposed. Proceedings of the 2023 4th International Symposium on Artificial Intelligence for Medicine Science; Chengdu, China: Association for Computing Machinery; 2024. p. 1036–42.

[13] Tan Y, Zhang Z, Li M, Pan F, Duan H, Huang Z, et al. MedChatZH: A tuning LLM for traditional Chinese medicine consultations. Comput Biol Med. 2024;172:108290. 38503097 10.1016/j.compbiomed.2024.108290

[14] Hua R, Dong X, Wei Y, Shu Z, Yang P, Hu Y, et al. Lingdan: enhancing encoding of traditional Chinese medicine knowledge for clinical reasoning tasks with large language models. J Am Med Inform Assoc. 2024;31(9):2019–29. 39038795 10.1093/jamia/ocae087 PMC11339528

[15] Dou Y, Huang Y, Zhao X, Zou H, Shang J, Lu Y, et al. ShennongMGS: An LLM-based Chinese Medication Guidance System. 2024.

[16] Dai Y, Shao X, Zhang J, Chen Y, Chen Q, Liao J, et al. TCMChat: A generative large language model for traditional Chinese medicine. Pharmacol Res. 2024;210:107530. 39617279 10.1016/j.phrs.2024.107530

[17] Liu C, Sun K, Zhou Q, Duan Y, Shu J, Kan H, et al. CPMI-ChatGLM: parameter-efficient fine-tuning ChatGLM with Chinese patent medicine instructions. Sci Rep. 2024;14(1):6403. 38493251 10.1038/s41598-024-56874-w PMC10944515

[18] Su Y, Hu X, Ma S, Zhang Y, Abudukelimu A, Halidenmu A. A review of artificial intelligence in traditional Chinese medicine diagnosis and treatment. Comput Eng Appl. 2024;60(16):1–18.

[19] Li X, Chen S, Meng M, Wang Z, Jiang H, Hao Y. Research progress and implications of the application of large language model in shared decision-making in China’s healthcare field. Front Public Health. 2025;13:1605212. 40709042 10.3389/fpubh.2025.1605212 PMC12287014

[20] Yip HF, Li Z, Zhang L, Lyu A. Large language models in integrative medicine: progress, challenges, and opportunities. J Evid Based Med. 2025;18(2):e70031. 40384541 10.1111/jebm.70031 PMC12086751

[21] Meng Q, Mi Y, Wang F, Guo H, Yang Y, Liu Y, et al. Intelligent technology leads the transformation of traditional Chinese medicine: large models and virtual cells aid modern analysis of stroke treatment. Pharmacol Res. 2025;221:107953. 40953761 10.1016/j.phrs.2025.107953

[22] Guo Y, Wang H, Ren X, Wang T, Chen W, Xu Z, et al. Can GPTs accelerate the development of intelligent diagnosis and treatment in traditional Chinese medicine? A survey and empirical analysis. J Evid Based Med. 2025;18(1):e70004. 39989008 10.1111/jebm.70004

[23] Shataer D, Cao S, Liu X, Aierken K, Bhattacharya P, Sinha A, et al. Application of large language models in traditional Chinese medicine: a state-of-the-art review. Am J Chin Med. 2025;53(4):973–97. 40582721 10.1142/S0192415X25500375

[24] Wang L, Tang K, Wang Y, Zhang P, Li S. Advancements in artificial intelligence-driven diagnostic models for traditional Chinese medicine. Am J Chin Med. 2025;53(3):647–73. 40374369 10.1142/S0192415X25500259

[25] Tricco AC, Lillie E, Zarin W, O’Brien KK, Colquhoun H, Levac D, et al. PRISMA Extension for scoping reviews (PRISMA-ScR): checklist and explanation. Ann Intern Med. 2018;169(7):467–73. 30178033 10.7326/M18-0850

[26] Yang G, Liu X, Shi J, Wang Z, Wang G. TCM-GPT: efficient pre-training of large language models for domain adaptation in traditional Chinese medicine. Comput Methods Programs Biomed Update. 2024;6:100158.

[27] Chin O, Jamil NS, Zainudin Z, Hitam NA, Ibrahim N, Sa'ahiry AHA, editors. OYEN: A User-Centric LLM-Based Bilingual Healthcare Chatbot. 2024 5th International Conference on Artificial Intelligence and Data Sciences (AiDAS); 2024 3–4 Sept. 2024.

[28] Zhang J, Yang S, Liu J, Huang Q. AIGC-empowered revitalization of TCM ancient texts: construction of Huang-Di large language model. Libr Tribune. 2024;44(10):103–12.

[29] Hai J, Wang R, Yuan L, Zhang K, Deng W, Xiao Y, et al. Exploration and practice of constructing retrieval-augmented question answering system for Traditional Chinese Medicine standard knowledge. Data Analysis and Knowledge Discovery. 2024.

[30] Zhang Y, Li H, Lang X, Zhou Z, Ling Y, Wang Z. Construction of Traditional Chinese Medicine question answering large language model based on retrieval-augmented generation technology. J Nanjing Univ Tradit Chin Med. 2024;40(12):1375–82.

[31] Wang Z, Li K, Ren Q, Yao K, Zhu Y, editors. Traditional Chinese Medicine Formula Classification Using Large Language Models. 2023 IEEE International Conference on Bioinformatics and Biomedicine (BIBM); 2023: IEEE.

[32] Haoyu T, Kuo Y, Xin D, Chenxi Z, Mingwei Y, Hongyan W, et al. TCMLLM-PR: evaluation of large language models for prescription recommendation in traditional Chinese medicine. Digit Chin Med. 2024;7(4):343–55.

[33] Zhang H, Wang X, Han L, Li Z, Chen Z, Chen Z. Research on question answering system integrating large language models with knowledge graphs. J Front Comput Sci Technol. 2023;17(10):2377–88.

[34] Ji X, Wang X, Zhang H, Meng Z, Zhang J, Zhuang P, et al. Research on knowledge enhancement methods for Traditional Chinese Medicine large language models. J Front Computer Sci Technol. 2024;18(10):2616–29.

[35] Yan Y, Ma T, Li R, Zheng X, Shan G, Li C. JingFang: A Traditional Chinese Medicine Large Language Model of Expert-Level Medical Diagnosis and Syndrome Differentiation-Based Treatment. arXiv preprint arXiv:250204345. 2025.

[36] Zhang H, Wang X, Meng Z, Chen Z, Zhuang P, Jia Y, et al. Qibo: A large language model for traditional chinese medicine. arXiv preprint arXiv:240316056. 2024.

[37] Yu S, Xu X, Xu F, Li L. Enhancing the Traditional Chinese Medicine Capabilities of Large Language Model through Reinforcement Learning from AI Feedback. arXiv preprint arXiv:241100897. 2024.

[38] Wei S, Peng X, Wang Y-f, Si J, Zhang W, Lu W, et al. BianCang: A Traditional Chinese Medicine Large Language Model. arXiv preprint arXiv:241111027. 2024. 10.1109/JBHI.2025.3612415 40991600

[39] Chen Y, Xiao Q, Yi J, Chen J, Wang M. Intelligent Understanding of Large Language Models in Traditional Chinese Medicine Based on Prompt Engineering Framework. arXiv preprint arXiv:241019451. 2024.

[40] Liu Y, Luo S, Zhong Z, Wu T, Zhang J, Ou P, et al. Hengqin-RA-v1: Advanced Large Language Model for Diagnosis and Treatment of Rheumatoid Arthritis with Dataset based Traditional Chinese Medicine. arXiv preprint arXiv:250102471. 2025.

[41] Zhou Z, Yang T, Hu K, editors. Traditional chinese medicine epidemic prevention and treatment question-answering model based on llms2023: IEEE.

[42] Zhou X, Dong X, Li C, Bai Y, Xu Y, Cheung KC, et al., editors. TCM-FTP: Fine-Tuning Large Language Models for Herbal Prescription Prediction2024: IEEE.

[43] Yue W, Wang X, Zhu W, Guan M, Zheng H, Wang P, et al. Tcmbench: A comprehensive benchmark for evaluating large language models in traditional chinese medicine. arXiv preprint arXiv:240601126. 2024.

[44] Yu P, Song K, He F, Chen M, Lu J. TCMD: A Traditional Chinese Medicine QA Dataset for Evaluating Large Language Models. arXiv preprint arXiv:240604941. 2024.

[45] Wang X, Chen GH, Song D, Zhang Z, Chen Z, Xiao Q, et al. Cmb: A comprehensive medical benchmark in chinese. arXiv preprint arXiv:230808833. 2023.

[46] Cao L, Xu L, Zhang Y, Zhang L, Fu Y, Jiang T. Standardized evaluation of large language models in Traditional Chinese Medicine. J Nanjing Univ Tradit Chin Med. 2024;40(12):1383–92.

[47] Zhang S, Dong L, Li X, Zhang S, Sun X, Wang S, et al. Instruction tuning for large language models: A survey. arXiv preprint arXiv:230810792. 2023.

[48] Naveed H, Khan AU, Qiu S, Saqib M, Anwar S, Usman M, et al. A comprehensive overview of large language models. arXiv preprint arXiv:230706435. 2023.

[49] Biesialska M, Biesialska K, Costa-Jussa MR. Continual lifelong learning in natural language processing: A survey. arXiv preprint arXiv:201209823. 2020.

[50] Yıldız Ç, Ravichandran NK, Punia P, Bethge M, Ermis B. Investigating continual pretraining in large language models: Insights and implications. arXiv preprint arXiv:240217400. 2024.

[51] Sun F-K, Ho C-H, Lee H-Y. Lamol: Language modeling for lifelong language learning. arXiv preprint arXiv:190903329. 2019.

[52] Dong G, Yuan H, Lu K, Li C, Xue M, Liu D, et al. How abilities in large language models are affected by supervised fine-tuning data composition. arXiv preprint arXiv:231005492. 2023.

[53] Lee H, Phatale S, Mansoor H, Lu KR, Mesnard T, Ferret J, et al. Rlaif: Scaling reinforcement learning from human feedback with ai feedback. 2023.

[54] Bai Y, Kadavath S, Kundu S, Askell A, Kernion J, Jones A, et al. Constitutional ai: Harmlessness from ai feedback. arXiv preprint arXiv:221208073. 2022.

[55] Schulman J, Wolski F, Dhariwal P, Radford A, Klimov O. Proximal policy optimization algorithms. arXiv preprint arXiv:170706347. 2017.

[56] Rafailov R, Sharma A, Mitchell E, Manning CD, Ermon S, Finn C. Direct preference optimization: Your language model is secretly a reward model. Adv Neural Inf Process Syst. 2023;36:53728–41.

[57] Lewis P, Perez E, Piktus A, Petroni F, Karpukhin V, Goyal N, et al. Retrieval-augmented generation for knowledge-intensive nlp tasks. Adv Neural Inf Process Syst. 2020;33:9459–74.

[58] Zhang Z, Zhang A, Li M, Smola A. Automatic chain of thought prompting in large language models. arXiv preprint arXiv:221003493. 2022.

[59] Hu EJ, Shen Y, Wallis P, Allen-Zhu Z, Li Y, Wang S, et al. Lora: Low-rank adaptation of large language models. ICLR. 2022;1(2):3.

[60] Dettmers T, Pagnoni A, Holtzman A, Zettlemoyer L. Qlora: Efficient finetuning of quantized llms. Adv Neural Inf Process Syst. 2023;36:10088–115.

[61] Swets JA. Measuring the accuracy of diagnostic systems. Science. 1988;240(4857):1285–93. 3287615 10.1126/science.3287615

[62] Davis J, Goadrich M, editors. The relationship between Precision-Recall and ROC curves. 2006.

[63] Chicco D, Jurman G. The advantages of the Matthews correlation coefficient (MCC) over F1 score and accuracy in binary classification evaluation. BMC Genomics. 2020;21:1–13. 10.1186/s12864-019-6413-7 PMC6941312 31898477

[64] Papineni K, Roukos S, Ward T, Zhu W-J, editors. Bleu: a method for automatic evaluation of machine translation. 2002.

[65] Lin C-Y, editor Rouge: A package for automatic evaluation of summaries. 2004.

[66] Banerjee S, Lavie A, editors. METEOR: An automatic metric for MT evaluation with improved correlation with human judgments. 2005.

[67] Zhang T, Kishore V, Wu F, Weinberger KQ, Artzi Y. Bertscore: Evaluating text generation with bert. arXiv preprint arXiv:190409675. 2019.

[68] Chang Y, Wang X, Wang J, Wu Y, Yang L, Zhu K, et al. A survey on evaluation of large language models. ACM Trans Intell Syst Technol. 2024;15(3):1–45.

[69] Lialin V, Deshpande V, Rumshisky A. Scaling down to scale up: A guide to parameter-efficient fine-tuning. arXiv preprint arXiv:230315647. 2023.

[70] Dai W, Zhang X, Wu R, Nie Y, editors. A large language model for traditional Chinese medicine based on Llama3. IET Conference Proceedings CP915; 2025: IET.

[71] Singhal K, Tu T, Gottweis J, Sayres R, Wulczyn E, Amin M, et al. Toward expert-level medical question answering with large language models. Nat Med. 2025;31(3):943–50. 39779926 10.1038/s41591-024-03423-7 PMC11922739

[72] Wei S, Peng X, Wang Y, Shen T, Si J, Zhang W, et al. Biancang: a traditional chinese medicine large language model. IEEE Journal of Biomedical and Health Informatics. 2025. 10.1109/JBHI.2025.3612415 40991600

[73] He J, Guo Y, Lam LK, Leung W, He L, Jiang Y, et al. Opentcm: a graphrag-empowered llm-based system for traditional chinese medicine knowledge retrieval and diagnosis. arXiv preprint arXiv:250420118. 2025.

[74] Li R, Ren G, Yan J, Zou B, Liu Q. Intelligent question answering system for traditional Chinese medicine based on BSG deep learning model: taking prescription and Chinese materia medica as examples. Digit Chin Med. 2024;7(1):47–55.

[75] Zhao C, Li G-Z, Wang C, Niu J. Advances in patient classification for traditional Chinese medicine: a machine learning perspective. Evid Based Complement Alternat Med. 2015;2015(1):376716. 26246834 10.1155/2015/376716 PMC4515265

