# Supplementary material for: Tuning and clinical application of large language models in Traditional Chinese Medicine: scoping review
Source: Chin Med. 2026 Feb 19;21:71. doi: 10.1186/s13020-026-01346-8 (PMC12922203; doi:10.1186/s13020-026-01346-8)
Supplement: Supplementary file 1 — Supplementary Material 1 [file 13020_2026_1346_MOESM1_ESM.docx]

**Table S1. Detailed Search Strategies for Each Database**

| **Database** | **Search Fields** | **Search Strategy** | **Results** |
| --- | --- | --- | --- |
| **PubMed** | Title/Abstract | (("large language model*"[Title/Abstract] OR "LLM*"[Title/Abstract] OR "Generative Pre-trained Transformer"[Title/Abstract] OR "GPT"[Title/Abstract] OR "generative AI"[Title/Abstract] OR "generative artificial intelligence"[Title/Abstract] OR "fine-tuning"[Title/Abstract] OR "reinforcement learning"[Title/Abstract] OR "direct preference optimization"[Title/Abstract] OR "DPO"[Title/Abstract] OR "supervised learning"[Title/Abstract] OR "proximal policy optimization"[Title/Abstract] OR "PPO"[Title/Abstract] OR "low-rank adaptation"[Title/Abstract] OR "LoRA"[Title/Abstract] OR "attention mechanism"[Title/Abstract] OR "pre-training"[Title/Abstract]) AND ("Traditional Chinese Medicine"[Title/Abstract] OR "TCM"[Title/Abstract] OR "Chinese medicine"[Title/Abstract] OR "Chinese herbal medicine"[Title/Abstract] OR "herbal formula*"[Title/Abstract] OR "acupuncture"[Title/Abstract] OR "moxibustion"[Title/Abstract] OR "cupping"[Title/Abstract] OR "Gua Sha"[Title/Abstract] OR "scraping"[Title/Abstract] OR "tuina"[Title/Abstract] OR "massage"[Title/Abstract] OR "external therapy"[Title/Abstract] OR "manipulation"[Title/Abstract])) | 805 records |
| **Web of Science** | Topic | TS=(("large language model*" OR "LLM*" OR "Generative Pre-trained Transformer" OR "GPT" OR "generative AI" OR "generative artificial intelligence" OR "fine-tuning" OR "reinforcement learning" OR "direct preference optimization" OR "DPO" OR "supervised learning" OR "proximal policy optimization" OR "PPO" OR "low-rank adaptation" OR "LoRA" OR "attention mechanism" OR "pre-training") AND ("Traditional Chinese Medicine" OR "TCM" OR "Chinese medicine" OR "Chinese herbal medicine" OR "herbal formula*" OR "acupuncture" OR "moxibustion" OR "cupping" OR "Gua Sha" OR "scraping" OR "tuina" OR "massage" OR "external therapy" OR "manipulation")) | 713 records |
| **IEEE Xplore** | All Metadata | ("large language model*" OR LLM* OR "Generative Pre-trained Transformer" OR GPT OR "generative AI" OR "fine-tuning" OR "reinforcement learning" OR "direct preference optimization" OR DPO OR "supervised learning" OR "proximal policy optimization" OR PPO OR "low-rank adaptation" OR LoRA OR "attention mechanism" OR "pre-training") AND ("Traditional Chinese Medicine" OR TCM OR "Chinese medicine" OR "Chinese herbal medicine" OR "herbal formula*" OR acupuncture OR moxibustion OR cupping OR "Gua Sha" OR scraping OR tuina OR massage OR "external therapy" OR manipulation) | 48 records |
| **ACM Digital Library** | Title, Abstract, Keywords | [[Title: "large language model"] OR [Title: LLM] OR [Title: "Generative Pre-trained Transformer"] OR [Title: GPT] OR [Title: "generative AI"] OR [Title: "fine-tuning"] OR [Title: "reinforcement learning"] OR [Title: LoRA] OR [Abstract: "large language model"] OR [Abstract: LLM] OR [Abstract: "Generative Pre-trained Transformer"] OR [Abstract: GPT] OR [Abstract: "generative AI"] OR [Abstract: "fine-tuning"] OR [Abstract: "reinforcement learning"] OR [Abstract: LoRA]] AND [[Title: "Traditional Chinese Medicine"] OR [Title: TCM] OR [Title: "Chinese medicine"] OR [Title: acupuncture] OR [Abstract: "Traditional Chinese Medicine"] OR [Abstract: TCM] OR [Abstract: "Chinese medicine"] OR [Abstract: acupuncture]] | 35 records |
| **arXiv** | Title, Abstract | (all:"large language model" OR all:LLM OR all:GPT OR all:"generative AI" OR all:"fine-tuning" OR all:"reinforcement learning" OR all:"direct preference optimization" OR all:DPO OR all:"supervised learning" OR all:PPO OR all:"low-rank adaptation" OR all:LoRA OR all:"attention mechanism" OR all:"pre-training") AND (all:"Traditional Chinese Medicine" OR all:TCM OR all:"Chinese medicine" OR all:acupuncture OR all:moxibustion OR all:cupping OR all:tuina OR all:massage) | 102 records |
| **CNKI**  (China National Knowledge Infrastructure) | Subject, Keyword, Abstract | (SU="大语言模型" OR SU="大模型" OR SU="生成式人工智能" OR SU="GPT" OR SU="微调" OR SU="强化学习" OR SU="监督学习" OR SU="低秩适应" OR SU="LoRA" OR SU="注意力机制" OR SU="预训练") AND (SU="中医" OR SU="中医药" OR SU="中药" OR SU="方剂" OR SU="针灸" OR SU="艾灸" OR SU="拔罐" OR SU="刮痧" OR SU="推拿" OR SU="按摩" OR SU="外治法" OR SU="手法") | 556 records |
| **Wanfang Database** | Title, Keyword, Abstract | (题名:"大语言模型" OR 题名:"大模型" OR 题名:"生成式人工智能" OR 题名:"GPT" OR 关键词:"大语言模型" OR 关键词:"大模型" OR 关键词:"微调" OR 关键词:"强化学习" OR 关键词:"LoRA" OR 关键词:"低秩适应" OR 关键词:"注意力机制" OR 关键词:"预训练") AND (题名:"中医" OR 题名:"中医药" OR 题名:"针灸" OR 题名:"推拿" OR 关键词:"中医" OR 关键词:"中医药" OR 关键词:"针灸" OR 关键词:"艾灸" OR 关键词:"拔罐" OR 关键词:"刮痧" OR 关键词:"推拿" OR 关键词:"按摩" OR 关键词:"外治") | 423records |
| **Total** | | | **2682 records** |

1. All searches were conducted on May 15, 2025.

2. Search date: May 15, 2025 for all databases.

3. Search date range: from database inception to May 2025.

4. No language restrictions were applied during the search process.

5. For Chinese databases (CNKI and Wanfang Database), search strategies were translated and adapted to fit database-specific syntax while maintaining semantic equivalence with the English search terms.

6. Boolean operators (AND, OR) and wildcards (*) were used where supported by the database platform.

**Table S2 Basic Information of Included Studies**

| **No.** | **First Author** | **Journal** | **Year** | **Region** | **Application** | **Research Objective** | **Results** |
| --- | --- | --- | --- | --- | --- | --- | --- |
| 1 | Hy Tian^1^ | Digital Chinese Medicine | 2024 | China | Formula recommendation | Develop LLM for TCM prescription recommendation | Significantly improved prescription recommendation; increased F1-score by 31.80% on TCM textbook dataset and 59.48% on Chinese Pharmacopoeia dataset |
| 2 | Xy Ji^2^ | Computer Science and Exploration | 2024 | China | Formula recommendation | Develop an LLM specialized for TCM prescription compatibility tasks and design an evaluation framework | Significant improvement over baseline models; BLEU-1 improved by up to 0.09, ROUGE-1 improved by up to 0.21 |
| 3 | Hy Zhang^3^ | Computer Science and Exploration | 2023 | China | Formula recommendation | Integrate LLM with knowledge graph to build a Q&A system in the TCM prescription field | System capable of information filtering, professional Q&A, and information extraction, better than baseline models with 70% accuracy in objective evaluations |
| 4 | Hua R^4^ | Journal of the American Medical Informatics Association | 2024 | China | Integrated D & T | ①Develop a foundational model for continuous pre-training on TCM knowledge  ②Develop a specialized model for diagnosis & treatment question answering and prescription recommendation | Excellent performance in Chinese patent medicine Q&A and prescription recommendation tasks, achieving an 18.39% improvement in Top@20 F1-score. |
| 5 | Dai Y^5^ | Pharmacological research | 2024 | China | Integrated D & T | Develop TCM LLM (entity extraction, diagnosis, prescription recommendation, ADMET prediction) | Herbal medicine recommendation accuracy reached 76.8%; surpassed GPT-3.5 in medical diagnosis and entity extraction tasks |
| 6 | Sb Wei^6^ | arXiv | 2024 | China | Integrated D & T | Develop TCM LLM (syndrome differentiation, diagnosis, case analysis, medical exam Q&A) | Excellent performance in TCM disease diagnosis and syndrome differentiation tasks; achieved 78.90% accuracy on TCMSD test dataset |
| 7 | S Yu^7^ | arXiv | 2024 | China | Integrated D & T | Develop TCM LLM to support physicians in initial and follow-up diagnoses | Significantly improved output quality; ROUGE-1 of 55.137 and ROUGE-L of 53.785, surpassing GPT-3.5 |
| 8 | Yr Chen^8^ | arXiv | 2024 | China | Integrated D & T | Design prompt engineering framework enhancing LLM performance in disease diagnosis, syndrome differentiation, and prescription recommendation | Overall performance improved by 11.64% on disease classification, syndrome recognition, and herbal recommendation tasks |
| 9 | Yh Yan^9^ | arXiv | 2025 | China | Integrated D & T | Develop specialized TCM diagnostic LLM for medical consultation, syndrome differentiation, and treatment recommendation | F1-score reached 0.8186 on TCM syndrome differentiation task; outperformed baseline models in medical consultation and multi-turn optimization |
| 10 | Hy Zhang^10^ | arXiv | 2024 | China | Integrated D & T | Develop TCM LLM (Qibo) for TCM consultation and syndrome differentiation | Rouge-L scores reached 0.72, 0.61, and 0.55 on three TCM NLP tasks; objective accuracy improved by 23% to 58% |
| 11 | Xz Zhou^11^ | 2024 -BIBM | 2024 | China | Specialized Disease D & T | ①Construct a dataset for digestive system diseases, DigestDS  ②Develop a large language model (LLM) for prescription and TCM dosage prediction | Achieved a prescription prediction F1-score of 0.8031, a dosage prediction NMSE of 0.0604, and a symptom-efficacy rating of 4.08 out of 5 |
| 12 | Zz Zhou^12^ | 2023-BIBM | 2023 | China | Specialized Disease D & T | Develop TCM LLM for epidemic disease prevention, knowledge Q&A, and prescription recommendation | BLEU-4: 44.02, ROUGE-L: 61.10; significantly better than baseline models in prescription generation |
| 13 | Ys Liu^13^ | arXiv | 2025 | China | Specialized Disease D & T | Develop TCM LLM specialized for rheumatoid arthritis diagnosis | Achieved 54% pass rate in TCM rheumatoid arthritis diagnosis, surpassing GPT-4 and other TCM LLMs |
| 14 | Jy Zhu^14^ | 2023-ISAIMS | 2024 | China | Comprehensive  Knowledge Consultation | ①Develop a TCM LLM, ZhongJing  ②Design a specialized evaluation method, TCMEval | Outperformed baseline models by 6.49 points on the TCMEval score and achieved an inference speed of 8 tokens per second on consumer-grade hardware |
| 15 | Tan, Y^15^ | Computers in Biology and Medicine | 2024 | China | Comprehensive  Knowledge Consultation | Develop dialogue-based LLM specialized for TCM consultation | Achieved BLEU-1 of 56.14 on webMedQA; ROUGE and multi-domain evaluation outperformed baseline models with enhanced professionalism and safety |
| 16 | O Chin^16^ | 2024-AiDAS | 2024 | Malaysia | Comprehensive  Knowledge Consultation | Develop bilingual TCM LLM for Q&A, query classification, information retrieval, and medical consultation | Successfully realized bilingual medical Q&A; query classification accuracy of 78%, supports Chinese, English, and mixed queries |
| 17 | Gx Yang^17^ | arXiv | 2023 | China | Comprehensive  Knowledge Consultation | Develop LLM applied in the TCM domain | Improved accuracy by 17.4% in TCM-EXAM tasks; excelled in training on TCM-specific corpus |
| 18 | Jl Hai ^18^ | Computer Science and Exploration | 2025 | China | Comprehensive  Knowledge Consultation | Develop TCM-standard knowledge Q&A system based on LLM and RAG | Efficiently answers diverse questions on TCM guidelines, herbal standards, information standards; outperformed baseline models |
| 19 | Jd Zhang^19^ | Library Tribune | 2024 | China | Comprehensive  Knowledge Consultation | Develop generative conversational LLM for ancient TCM literature (ancient literature Q&A, TCM diagnosis, health care) | Built ancient literature dataset; improved ancient literature knowledge Q&A, outperforming existing TCM LLMs; DPO enhanced accuracy, completeness, and fluency |
| 20 | Ym Zhang^20^ | Journal of Nanjing University of Traditional Chinese Medicine | 2024 | China | Comprehensive  Knowledge Consultation | Construct RAG-based TCM Q&A LLM to reduce hallucinations, enhancing accuracy and reliability | Accuracy over 90% for simple questions, F1-score above 0.766 for medium/hard questions; superior diversity and accuracy compared to base models |
| 21 | Liu C^21^ | scientific Reports | 2024 | China | Medication consultation | Develop specialized LLM for TCM drug instruction generation and recommendations | Outperformed comparative models in all evaluation metrics; P-Tuning v2 fine-tuning method better than LoRA |
| 22 | Yt Dou^22^ | ACM Transactions on Management Information Systems | 2024 | China | Medication consultation | ①Construct a drug knowledge graph  ②Develop a LLM for medication guidance and adverse reaction prediction. | Achieved a score of 94 in AI evaluation and 97 in expert evaluation, demonstrating outstanding performance in medication guidance and adverse reaction prediction, especially in scenarios involving polypharmacy. |
| 23 | Z Wang^23^ | 2023-BIBM | 2023 | China | Formula classification | Develop LLM for TCM prescription classification | Fine-tuned ChatGLM2-6b achieved 71% accuracy; significantly improved small-scale model performance |
| 24 | Wj Yue^24^ | arXiv | 2024 | China | Benchmark testing | Construct comprehensive benchmark (TCMBench) for evaluating LLMs in TCM domain | All evaluated LLMs failed TCMBench; GPT-4 performed best (accuracy 59.86%); models integrating TCM knowledge performed better at similar parameter scales |
| 25 | P Yu^25^ | arXiv | 2024 | China | Benchmark testing | Design TCMD for systematic evaluation | General-purpose LLMs outperformed medical and TCM-specific LLMs; Moonshot-v1-8k and InternLM2-Chat-20B with CoT performed best |
| 26 | Xd Wang^26^ | arXiv | 2024 | China | Benchmark testing | Develop integrated TCM-Western medicine LLM benchmark (CMB) | GPT-4 and some open LLMs achieved over 60% accuracy on CMB-Exam; significant accuracy variations between knowledge domains; HuatuoGPT-II performed best |
| 27 | Lu Cao ^27^ | Journal of Nanjing University of Traditional Chinese Medicine | 2024 | China | Benchmark testing | Develop benchmark dataset to objectively evaluate TCM knowledge and reasoning capabilities of LLMs | General LLMs slightly outperformed Chinese medical LLMs; all models had <60% accuracy on multiple-choice questions, indicating challenges in TCM domain |

Note: All studies are sorted according to application scenarios, and references are indicated in the "First Author" column. TCM：Traditional Chinese Medicine；LLM：large language model；SFT: Supervised Fine-Tuning; Lora: Low-Rank Adaptation; P-Tuning v2: Parameter Tuning v2; DPO: Direct Preference Optimization; RAG: Retrieval-Augmented Generation; BLEU: Bilingual Evaluation Understudy; ROUGE: Recall-Oriented Understudy for Gisting Evaluation; GLEU: Google’s Language Evaluation Utility; METEOR: Metric for Evaluation of Translation with Explicit Ordering; MAE: Mean Absolute Error; RMSE: Root Mean Squared Error; nDCG: normalized Discounted Cumulative Gain; MRR: Mean Reciprocal Rank (used for evaluating ranking); ROC-AUC: Receiver Operating Characteristic - Area Under Curve; NMSE: Normalized Mean Squared Error; BART: Bidirectional and Auto-Regressive Transformers; RAGAs: Retrieval-Augmented Generation Assistant; TCMD: A Traditional Chinese Medicine QADataset; CMB: A Comprehensive Medical Benchmark in Chinese.

**Table S3 Summary of tuning Conditions in TCM large language models (23 Studies)**

| **No.** | **First Author** | **Application Scenario** | **Base Model** | **Training and Fine-tuning Methods** | **Training Data** | **Evaluation Metrics** | **Model Comparisons** | **Evaluation Data** | **Open-source Link** |
| --- | --- | --- | --- | --- | --- | --- | --- | --- | --- |
| 1 | Hy Tian^1^ | Prescription recommendation | ChatGLM-6B | P-Tuning v2 | ①Four TCM textbooks  ②Pharmacopoeia of PRC ③Clinical case data | ① Precision@K ② Recall@K ③ F1-score@K | ① PTM(d), TCMPR, PresRecST ②ShenNong,Huatuo, HuatuoGPT, ChatGPT | 9prescription recommendation test sets | <https://github.com/2020MEAI/TCMLLM> |
| 2 | Xy Ji^2^ | Prescription recommendation | Chinese-LLaMA-7B/13B | ①Continuous Pre-training ②SFT ③RAG | ①8 types of continuous pretraining data^#1^ ②6 fine-tuning datasets^#2^ | ①BLEU-1  ②ROUGE-1  ③RAGAs  ④Human evaluation ^*1^ | ①Chinese-LLaMA ②HuatuoGPT ③Zhongjing | Prescription QA data from public sources (unspecified) | - |
| 3 | Hy Zhang^3^ | Prescription recommendation | ChatGLM-6B | ①P-Tuning v2 ②Prompt Engineering ③RAG | ①4 public medical datasets ^#3^ ②Web-scraped data ③LLM-generated QA/fine-tuning data | ①Accuracy ②Human evaluation^*2^ | ①ChatGLM-6B ②ChatGPT | ①50 prescription MCQs ②100 TCM QA problems | <https://github.com/zhangheyi1/llmkgqas-tcm/> |
| 4 | Hua R^4^ | Diagnostic assistance | Baichuan2-13B-Base | ①Continued Pre-training ②Qlora Fine-tuning ③Prompt Engineering | ①1,522 ancient TCM texts ② Modern textbooks ③2020 Pharmacopoeia ④39,122 clinical cases ⑤4 public datasets ^#4^ | ①Human evaluation (ancient text translation) ^*3^ ②Diagnosis QA (automatic + human) ^*4^ ③Prescription recommendation: Precision@K, Recall@K, F1@K | ①Lingdan-TCPM-Chat ②Baichuan2-TCPM-Chat ③ChatGPT ④Baichuan2-13B-Chat ⑤Baichuan2-13B-Base | ①20 translation samples ②Doctor-patient QA dialogues ③Outpatient case prescription test set | <https://github.com/TCMAI-BJTU/LingdanLLM> |
| 5 | Dai Y^5^ | Diagnostic assistance | Baichuan2-7B-Chat | ①Continued Pre-training ②full-parameter Fine-tuning | ①Standards and textbooks ②Crawled data from TCM sites ③500k literature abstracts ④6 public medical datasets^#5^ | ①Multiple-choice questions: Accuracy ②Reading comprehension: BLEU score, METEOR score, ROUGE score, BERTScore ③diagnosis: Precision, Recall, F1-score ④formula recommendation: MRR, Precision@K, nDCG ⑤ADMET prediction: MAE, RMSE, Accuracy, ROC-AUC | ①GPT-3.5-turbo，②Gemini-pro，③Bentsao-med，④Bentsao-literature，⑤BianQue2，⑥HuatuoGPT，⑦CMLM-zhongjing，⑧ChemProp，⑨MgBert，⑩DrugAssist | 480 entity samples; 500 each for MCQ, QA, case study, prescription, and ADMET prediction | https://github.com/ZJUFanLab/TCMChat |
| 6 | Sb Wei^6^ | Diagnostic assistance | ①Qwen2-7B/14B ②Qwen2.5-7B/14B | ①Continuous Pre-training ②Full-parameter fine-tuning | ①Pre-training: 460M tokens (1.5M instances)  ②Fine-tuning: 228M tokens (720k instances) | ①Syndrome differentiation accuracy ②Disease diagnosis accuracy ③Medical exam accuracy ④Human evaluation^*5^ | ①8 general LLMs ②3 TCM-specialized LLMs ③12 general medical LLMs ④6 internal versions | ①TCMSD  ②TCMDD  ③MLEC-QA、CMB ④50 complex TCM cases | <https://github.com/QLU-NLP/BianCang> |
| 7 | S Yu^7^ | Diagnostic assistance | ①GLM-4-9B  ②Llama-3-8B  ③Qwen2-7B  ④DeepseekMOE-16B | ①Lora Fine-tuning ②DPO | ①71 initial visit and 188 follow-up cases ②Open-source datasets (unspecified) | ①ROUGE，②BLEU-4，③Precision，④Recall, ⑤F1-Score，⑥BERT-Score | GPT-3.5-turbo | 208 prescription test cases 100 validation cases | - |
| 8 | Yr Chen^8^ | Diagnostic assistance | Pretrained LLM (unspecified) | Prompt Engineering | TCM-specific and general NLP tasks (not clearly specified) | Accuracy | - | - | - |
| 9 | Yh Yan^9^ | Diagnostic assistance | Qwen2.5-7B-Instruct | ①Lora Fine-tuning ②RAG ③Prompt Engineering | ①43,000 clinical records ②100 medical consultation dialogues | ①Syndrome: Accuracy, Precision, Recall, F1 ②Consultation: Human evaluation^*6^ | ①Comparison of the dialectical accuracy among 6 LLMs（GPT-4o、Qwen-max、Sun Simiao-7B、Zhong Jing-7B、Ming Yi-7B、Shen Nong-7B） ②Comparison of medical consultation capabilities among 4 LLMs（Sun Simiao、Shen Nong、Ming Yi、Zhong Jing） | ①8,699 test cases ②100 medical consultation samples | - |
| 10 | Hy Zhang^10^ | Diagnostic assistance | Chinese-LLaMA-7B/13B | ①Continuous Pre-training ②Full-parameter fine-tuning ③RAG | ①8 types of pretraining data^#6^ ②Fine-tuning sets: ChatMed-TCM，CMtMedQA | ①Accuracy ②Rouge-L  ③Human evaluation^*7^ | ①ChatGPT，②Chinese-LLaMA，③BenTsao，④DoctorGLM，⑤HuatuoGPT，⑥ZhongJing | ①Test dataset（3,175 exam questions, 517 prescriptions, 689 syndrome, 475 reading） ②Human evaluation：150 TCM QAs | - |
| 11 | Xz Zhou^11^ | Diagnostic assistance (specific disease) | ①Chinese-Alpaca-Plus-7B ②ShenNong | ①Lora Fine-tuning ②Prompt Engineering | 18,953 digestive disease clinical cases | ①Precision，②Recall，③F1-Score，④NMSE，⑤Human evaluation（Expert evaluation）^*8^ | ①PTM，②TCMPR，③KDHR，④PresRecST，⑤Mengzi (T5-base)，⑥GPT-3.5，⑦GPT-4.0 | 2,057 test samples | - |
| 12 | Zz Zhou^12^ | Diagnostic assistance (specific disease) | ChatGLM | Lora Fine-tuning | ①194 ancient TCM texts ②TCM epidemic prevention knowledge graph | ①BLEU-4  ②ROUGE-L ③METEOR | ①Chinese-alpaca-plus ②ChatGLM ③ChatGLM2 ④ChatGPT | 50 prescription generation questions | - |
| 13 | Ys Liu^13^ | Diagnostic assistance (specific disease) | LLaMA-7B Huatuo2 | ①Lora Fine-tuning ②RAG | ①TCM books ②Journals and theses ③Practice exam questions ④Clinical records | ①TCM practitioner exam：Accuracy ②Human evaluation^*9^ | ①Baichuan，②ChatYuan，③Huatuo-2-7B，④Linly，⑤ChatGLM-6B，⑥ERNIE，⑦GPT（2.5、2.5+、3.5、3.5+、4、4o） | ①TCM exam questions ②ancient TCM texts | - |
| 14 | Jy Zhu^14^ | General knowledge consultation | Chinese-LLaMA2-7B | ①Lora Fine-tuning ②Prompt Engineering | 1,125,564 QAs from ShenNong TCM dataset | ①TCMEval  ②Rouge  ③BLEU | ①LinkSoul/Chinese-LLaMA2-7B ②Seeledu/Chinese-LLaMA2-7B ③Atom-7B | ①200 TCMEval questions ②300 general QA samples | - |
| 15 | Tan, Y^15^ | General knowledge consultation | Baichuan-7B | ①Continued Pre-training ②full-parameter fine-tuning | ①1000+ classic TCM texts ②9 public medical datasets ^#7^ | ①BLEU，②GLEU，③ROUGE，④Reward model score | ①GPT-3.5-turbo，②HuatuoGPT，③ChatGLM-Med，④LLaMA-Med，⑤BenTsao | webMedQA dataset | <https://github.com/tyang816/MedChatZH> |
| 16 | O Chin^16^ | General knowledge consultation | Llama2(quantized) | ①RAG ②Prompt Engineering | - | ①Accuracy，②Precision，③Recall，④F1-Score | Llama2 | - | - |
| 17 | Gx Yang^17^ | General knowledge consultation | BLOOM-7B | Lora Fine-tuning | ①Baidu Baike, Wikipedia  ②TCM-EXAM(6,325 MCQs)  ③7,783 clinical cases | Accuracy | BLOOM-7B-base BLOOM-7B-Random | 421 MCQs, 300 case records | - |
| 18 | Jl Hai ^18^ | General knowledge consultation | GPT-3.5 | Retrieval-Augmented Generation（RAG） | 940 TCM industry standards | ①Precision ②Recall ③F1-Score | ①BaiChuan ②Gemma ③Qwen-14B ④Qwen2-7B | ①3,000 standard TCM QA pairs ②13,000 TCM literature QA pairs | - |
| 19 | Jd Zhang^19^ | General knowledge consultation | Ziya-LLaMA-13B-v1 | ①Continuous Pre-training ②Lora Fine-tuning ③DPO | ①22 TCM textbooks, web data ②General dialogue data  ③504,372 ancient text records | ①BLEU-4  ②ROUGE-1/2/L ③Human evaluation^*10^ | ①Qwen  ②ChatGPT(gpt-4)  ③ShengNong-TCMTCMLLM | 10,000 test QA pairs | <https://github.com/Zlasejd/Huang-DI> |
| 20 | Ym Zhang^20^ | General knowledge consultation | ChatGLM2-6B | ①P-Tuning v2 ②Prompt Engineering ③RAG | ①TCM classical literature ②Empirical prescriptions from senior TCM doctors ③QA fine-tuning dataset | ①Accuracy ②Recall ③F1-Score ④Human evaluation^*11^ | ChatGLM2-6B | Test dataset | - |
| 21 | Liu C^21^ | Medication consultation | ChatGLM-6B | ①P-Tuning v2 ②Lora Fine-tuning | ①TCM drug guideline ②Aliyun TCM NER dataset ③Clinical cases | ①BLEU，②ROUGE，③BART-Score，④Human evaluation ^*12^ | ①Chinese-LLaMA-7B ②Chinese-Alpaca-7B ③Qwen-7B ④Baichuan-7B | Test dataset | <https://github.com/liucann/CPMI-ChatGLM> |
| 22 | Yt Dou^22^ | Medication consultation | ChatGLM-6B | P-Tuning v2 | ①8 medical public datasets^#8^ ②22,327 clinical dialogues ③13,020 medical QAs | Human evaluation^*13^ | ①ChatGPT ②NewBing ③ChatGLM ④HuatuoGPT ⑤BentsaoGPT | ①50 drug questions ②50 real-case medical Qs | <https://github.com/pengslab/ShennongGPT> |
| 23 | Z Wang^23^ | Prescription classification | ChatGLM-6b ChatGLM2-6b | ①Prompt Engineering ②Lora Fine-tuning | ①Formula coding guidelines ②National insurance drug list  ③ TCM formula textbook ④ 2,617 formulas | Accuracy | ①ChatGLM-6b ②ChatGLM2-6b ③ChatGLM-130b  ④InternLM-20b ⑤wuChatGPT | 208 test prescriptions 100 validation samples | - |

Note: All studies are sorted based on items 1–23 in Table 1, and references are indicated in the "First Author" column.

#(1–8): Specific datasets used in different studies are detailed in Supplementary Table 1, and references to these are marked with "#" in this table.

*(1–13): Specific manual evaluation methods used in different studies are detailed in Supplementary Table 2, and references to these are marked with "" in this table.

TCM: Traditional Chinese Medicine; LLM: Large Language Model; P-Tuning v2: Parameter Tuning v2; SFT: Supervised Fine-Tuning; RAG: Retrieval-Augmented Generation; Lora: Low-Rank Adaptation; DPO: Direct Preference Optimization; BLEU: Bilingual Evaluation Understudy; ROUGE: Recall-Oriented Understudy for Gisting Evaluation; GLEU: Google’s Language Evaluation Utility; METEOR: Metric for Evaluation of Translation with Explicit Ordering; MAE: Mean Absolute Error; RMSE: Root Mean Squared Error; nDCG: normalized Discounted Cumulative Gain; MRR: Mean Reciprocal Rank; ROC-AUC: Receiver Operating Characteristic - Area Under Curve; NMSE: Normalized Mean Squared Error; BERT: Bidirectional Encoder Representations from Transformers; BART: Bidirectional and Auto-Regressive Transformers.

**Table S4 Summary of TCM LLMs Benchmark Evaluations (4 Studies)**

| **No.** | **Author** | **Data Content** | **Question Type** | **Domain Distribution** | **Evaluation Metrics** | **Evaluated Models** |
| --- | --- | --- | --- | --- | --- | --- |
| 1 | Wj Yue^24^ | ①TCM-ED dataset (TCMLE: 5,473 Q&A pairs)  ②TMNLI dataset (9,788 standard analysis exam questions, 29,497 constructed NLP inference samples) | A1: Single-sentence single-choice questions A2: Case-based single-choice questions A3: Shared-case multiple questions B1: Multiple-choice questions | Basic TCM theory, TCM diagnostics, Chinese materia medica, formulas, Huangdi Neijing, Treatise on Cold Damage, Essential Prescriptions of the Golden Cabinet, febrile diseases, TCM internal medicine, surgery, gynecology, pediatrics, acupuncture, internal medicine, diagnostics, infectious diseases, medical ethics, health law | ①Accuracy，②ROUGE-1，③ROUGE-L，④SARI，⑤BertScore，⑥BartScore，⑦TCMScore，⑧Human evaluation | ①GPT-4，②ChatGPT，③ChatGLM，④Chinese LlaMa ⑤HuaTuo，⑥ZhongJing-TCM |
| 2 | P Yu^25^ | TCMLE exam questions (Training set: 2,851 QAs; Test set: 600 QAs) | A1: Single-sentence single-choice A2: Summary-based clinical case single-choice A3: Shared-case single-choice B1: Shared-option multiple-choice | ① Basic TCM (23.33%): Basic theory, diagnostics, materia medica, formulas ② TCM classics (3.33%): Neijing, Cold Damage, Golden Cabinet, febrile diseases ③ Clinical TCM practice (50%): Internal, surgery, gynecology, pediatrics, acupuncture ④ Western medicine (20%): Internal medicine, diagnostics, infectious diseases ⑤ Humanities (3.33%): Ethics, health law | Sub-domain comparison across field, question type, prompting style ① Accuracy ② Consistency | ① General LLMs: ChatGPT, Moonshot-v1-8k, ChatGLM, Baichuan, QwenChat, InternLMChat, AquilaChat, Vicuna  ② Medical LLMs: ChatMed  ③ TCM LLMs: ShenNong, HuaTuo |
| 3 | Xd Wang^26^ | 280,839 multiple-choice questions across 6 categories, in two parts: ① CMB-Exam: Exam-style questions ② CMB-Clin: Real-case complex diagnostic problems | ① Multiple-choice questions ② Complex case-based diagnosis (multi-turn dialogue simulating doctor-patient interaction) | ① Physicians: 124,926 ② Nursing: 16,919 ③ Medical technology: 27,004 ④ Pharmacists: 33,354 ⑤ Academic exams: 62,271 ⑥ Graduate entrance: 16,365 | ① CMB-Exam: Accuracy ② CMB-Clin: Expert & GPT-4 evaluation (fluency, relevance, completeness, medical expertise; each scored 1–5) | ① Medical models: Huatuo GPT-II, ChatMed-Consult, MedicalGPT, ChatGLM-Med, DoctorGLM, Bianque-2, Bencao, IvyGPT, Sunsimiao, DISC-MedLLM. ② General models: ChatGLM3, Baichuan2, Qwen, Yi, Deepseek, Mistral, Internlm. ③ Commercial: ChatGPT, GPT-4, ShukunGPT, GLM-Med |
| 4 | Lu Cao^27^ | 31,197 exam questions from: ① Real TCM exam papers (Practitioner: 2007–2022; Graduate: 1991–2019) ② 6 official TCM textbooks | ① Answer prediction: 29,506 questions (single & multiple choice) ② Answer inference: 1,691 open-ended Q&A | 13 disciplines including basic theory, diagnostics, materia medica, formulas, acupuncture, internal medicine, surgery, gynecology, pediatrics, classics, law, ethics, humanities | ① Answer prediction: Accuracy, F1 score ② Answer inference: BLEU, ROUGE | ① General models: GPT-3.5, ChatGLM3-6B, Baichuan-13B-Chat  ② Chinese medical models: PULSE, BenTsao, HuatuoGPT2, BianQue2, ShenNong |

Note: The 4 studies are sorted based on items 24-27 in Table 1, and references are indicated in the "First Author" column. TCM: Traditional Chinese Medicine ; TCMLE: Traditional Chinese Medicine Licensing Exam; NLP: Natural Language Processing; ROUGE: Recall-Oriented Understudy for Gisting Evaluation; SARI: System for Automatic Readability Assessment; BLEU: Bilingual Evaluation Understudy (a metric for evaluating machine translation); BERT: Bidirectional Encoder Representations from Transformers; BART: Bidirectional and Auto-Regressive Transformers.

**Table S5 . Overview of large language models Tuning methods in TCM Domain**

| **Technique** | **Advantages and Applications in TCM** |
| --- | --- |
| CPT | Enables deeper domain knowledge integration through additional training on specialized data; particularly beneficial for TCM with its unique terminology system, enhancing comprehension of classical literature and theoretical correlations while establishing a solid foundation for subsequent fine-tuning |
| RAG | Incorporates external knowledge retrieval during generation to reduce hallucinations and improve accuracy; especially valuable for TCM's extensive and evolving knowledge system, effectively addressing specialist knowledge requirements for rare prescriptions and historical medical cases |
| PE | Guides output through carefully designed instructions without parameter modification; allows creation of templates aligned with TCM diagnostic workflows from "four examinations" to "syndrome differentiation and treatment", better simulating TCM clinical reasoning and rapidly adapting to various clinical scenarios |
| Fine-tuning |  |
| SFT | Adjusts parameters using annotated data to guide expected outputs; applicable for TCM syndrome differentiation and prescription recommendation tasks, though effectiveness highly depends on the quality of professional training data |
| RLHF | Optimizes outputs through reward models to align with human expectations; enhances prescription safety and adherence to TCM principles such as "Jun-Chen-Zuo-Shi" compatibility, despite higher technical implementation complexity |
| DPO | Simplifies RLHF workflow through direct optimization based on human preferences; well-suited for TCM's emphasis on experiential knowledge, incorporating professional physician feedback to ensure outputs conform to clinical practice standards |
| **Parameter Scope** | **Characteristics and advantages** |
| Full | Achieves optimal performance improvements by updating all model parameters; delivers superior TCM professional capabilities when sufficient computational resources are available, suitable for well-resourced research teams |
| LoRA | Implements parameter-efficient tuning through low-rank decomposition matrices; significantly reduces hardware barriers for TCM-LLM tuning while maintaining performance comparable to full fine-tuning, enabling broader research participation |
| QLoRA | Further reduces memory requirements by quantizing models before applying LoRA; enables large-scale model tuning in single-GPU environments, suitable for tasks requiring substantial parametric capacity |
| P-Tuning | Optimizes continuous prompt embeddings while keeping pre-trained parameters frozen; maintains reasonable performance for basic TCM knowledge tasks with minimal computational resources |
| P-Tuning v2 | Enhances performance by adding trainable prompt tokens across all transformer layers; improves complex TCM reasoning tasks with minimal parameter updates, effective for syndrome differentiation and treatment planning |

Note: CPT: Continued Pre-Training, DPO: Direct Preference Optimization, LLMs: Large Language Models, LoRA: Low-Rank Adaptation, PE: Prompt Engineering, QLoRA: Quantized Low-Rank Adaptation, RAG: Retrieval-Augmented Generation, RLHF: Reinforcement Learning from Human Feedback, SFT: Supervised Fine-Tuning, TCM: Traditional Chinese Medicine.

**Table S6 The specific datasets of different studies**

| **No.** | **datasets** |
| --- | --- |
| #1 | ① Medical textbooks (38.1M), ② TCM textbooks (40.6M), ③ TCM medical journals (317.0M), ④ TCM case studies (49.8M), ⑤ Other ancient TCM texts (165.0M), ⑥ TCM encyclopedia (563.0M), ⑦ TCM literature (50.4M), ⑧ Syndrome differentiation materials (50.4M), ⑨ TCM formulations (13.5M) |
| #2 | ① CMtMedQA (Traditional Chinese Medicine Q&A), ② ChatMed (extracted from TCM knowledge graphs), ③ Prescription Entity Recognition dataset, ④ TCM-RC (TCM literature dataset), ⑤ Simple Medical Dialogue (TCM general Q&A), ⑥ TCM-SD (TCM syndrome differentiation dataset) |
| #3 | ① MedDialog (medical dialogue dataset), ② CBLUE (8 TCM-related datasets), ③ COMETA (20,000 biomedical entities), ④ CMeKG (Chinese medical knowledge graph) |
| #4 | ①ChatMed_Consult、②CMtMedQA、③qizhenGPT、④WuDaoTCM |
| #5 | ① ETCM database (herbs and formulations data), ② Alibaba Tianchi TCM reading comprehension (18,478 entries), ③ TCM-NER entity recognition data (2,480 entries), ④ ShenNong_TCM_Dataset (110,000 entries), ⑤ Herb2.0 (herbal molecular data), ⑥ PharmaBench (ADMET data) |
| #6 | ① Medical books (38.1M), ② TCM books (40.6M), ③ TCM Compendium (317M), ④ Medical specialties (49.8M), ⑤ Other ancient texts (165M), ⑥ TCM encyclopedia (563M), ⑦ TCM reading comprehension (40.2M), ⑧ TCM syndrome differentiation (50.4M), ⑨ TCM prescriptions (13.5M) |
| #7 | ① Medical books (belle-3.5M), ② Medical dialogue data (medical), ③ Multi-turn medical dialogues (medical-dialogue), ④ Taiwan medical conversation data (medical-qa-instruction-zhtw), ⑤ Belle multi-turn dialogues (multiturn_chat_0.8M), ⑥ Huatuo project medical consultations (huatuo/consultation), ⑦ Huatuo project Q&A data (huatuo/encyclopedia_qa), ⑧ Huatuo project knowledge graph Q&A (huatuo/knowledge_graph_qa), ⑨ Huatuo project fine-tuning data (huatuo/sft-data-v1) |
| #8 | ① FDA-related data (21,508 entries), ② PubMed biomedical literature (79,000 entries), ③ DrugBank database (1,888,733 entries), ④ Drugs.com medication information (24,000 entries), ⑤ UpToDate clinical information (29,800 entries), ⑥ PubMedQA biomedical Q&A dataset (273,500 entries), ⑦ ChatMed patient online queries (110,113 entries), ⑧ Med-ChatGLM patient case reports/studies (7,622 entries) |

**Table S7 The specific human evaluation methods in different studies**

| No. | **Evaluation methods** |
| --- | --- |
| *1 | **Safety**: (1) Model must provide comprehensive professional explanations; (2) Prescribed formulations must not contain obvious contraindications; (3) Model must not answer privacy-related questions; (4) Model must refuse to answer potentially harmful questions.  **Accuracy**: (1) Prescribed formulations must demonstrate corresponding therapeutic effects; (2) Provide accurate explanations for recommended formulations.  **Fluency**: (1) Responses should be linguistically fluent with clear meaning; (2) No irrelevant or contradictory information; (3) Content should be friendly and benevolent.  **Precision rate**: Calculated as the proportion of herbs in the model's formulation that also appear in the expert-created formulation, relative to the total number of herbs in the expert formulation. |
| *2 | Three TCM experts evaluated responses from different models (professional Q&A systems, ChatGLM, and ChatGPT). Using 100 questions (general and specialized), experts assessed answer quality based on professionalism, accuracy, and completeness, selecting the most satisfactory response. Results were quantified as satisfaction rates for each model. |
| *3 | **Sample selection**: 20 classic text translation examples from TCM ancient literature **Models compared**: ChatGPT-3.5, Baichuan2-13B-Chat, Llama2-13B-Chat, ChatGLM **Expert panel**: 3 TCM researchers **Evaluation dimensions**:   - Accuracy (faithful transmission of original meaning) - Fluency (readability of translated text) - Creativity (preservation of literary and cultural expressive elements) - Cultural heritage (retention of ancient TCM cultural context and essence) |
| *4 | **TCPM-Chat evaluation**  **Test case selection**: Multiple simulated patient-doctor dialogue scenarios  **Comparison models**: Lingdan-TCPM-Chat vs. Baichuan2-TCPM-Chat, ChatGPT, and Baichuan2-13B-Chat  **Capability testing**: Evaluates whether models can inquire about relevant symptoms based on initial presentation, analyze symptoms using TCM theory, recommend appropriate Chinese patent medicines, and explain efficacy and precautions. |
| *5 | **Dataset**: BC-Analytical (50 complex TCM cases)  **Evaluation dimensions**: Professionalism, fluency, safety  **Metrics**: Win rate, tie rate, loss rate  **Evaluators**: Hospital physicians |
| *6 | TCM experts scored based on four dimensions (proactivity, accuracy, practicality, overall effect), with 10 points maximum per dimension (40 points total)  **Comparison models**: Sun Simiao, Shen Nong, Ming Yi, Zhong Jing |
| *7 | **Metrics**: Win rate, tie rate, and loss rate across safety, professionalism, and fluency dimensions  **Method**: 150 TCM-related questions, scored by GPT-4; complex safety assessments validated by medical expert evaluations |
| *8 | Random selection of 20 samples from test set, evaluated by 5 TCM experts Each prescription scored on symptom-efficacy and medicinal compatibility dimensions (0-5 points each, 10 points total) |
| *9 | **Expert evaluation**: Assessment of model-generated diagnoses and prescriptions  **Evaluation dimensions**: Coverage of four diagnostic methods, accuracy of syndrome differentiation |
| *10 | 10 TCM experts participated in scoring, with evaluation indicator weights determined through three rounds of Delphi method. Scoring across three dimensions (0-10 points):   - Content accuracy: Evaluating whether generated answers accurately reflect knowledge from TCM ancient texts - Linguistic fluency: Assessing whether generated content is grammatically sound with natural language expression - Answer completeness: Determining whether responses comprehensively cover all important aspects of the question |
| *11 | Experts evaluated 20 sample questions (four versions each) on five aspects (1 point per dimension, 5 points per question, 100 points maximum):   - Score1: Whether generated questions are highly similar - Score2: Whether answers to generated questions are identical - Score3: Whether questions can be answered from the document - Score4: Whether questions are coherent - Score5: Whether questions have misleading tendencies |
| *12 | Random selection of 20 herbal medicine recommendation questions, evaluated by 5 TCM practitioners across three dimensions (safety, usability, smoothness) using a 3-point scale: 1 (unacceptable) - 2 (acceptable) - 3 (good)   - Safety: Assessing whether model-generated content might mislead users or endanger health - Usability: Evaluating whether content demonstrates professional knowledge - Smoothness: Assessing the model's text generation capabilities as an LLM |
| *13 | Evaluation through 50 basic pharmaceutical knowledge questions and 50 actual case variation questions, scored by GPT-4 and human medical professionals across 5 dimensions:  **Query comprehension**   - - High: Accurately grasps core question intent including drug names, uses, question type   - Medium: Basically understands questions but may miss details   - Low: Misinterprets key points or provides irrelevant answers   **Situation analysis**   - - High: Comprehensive understanding of medical context, considering patient conditions and drug properties   - Medium: Partially recognizes contextual importance but analysis lacks depth   - Low: Ignores important contextual factors, providing overly generic answers   **Medication advice rationality**   - - High: Evidence-based recommendations following clinical principles and considering drug characteristics   - Medium: Basically reasonable suggestions but lacking depth or specificity   - Low: Recommendations inconsistent with medical standards or presenting risks   **Adverse reaction identification**   - - High: Comprehensive identification of potential risks including common and rare side effects, especially for polypharmacy   - Medium: Basic side effects noted but not comprehensive   - Low: Important adverse effect risks overlooked, insufficient information   **Description comprehensiveness**   - - High: Thorough, well-structured answers covering all aspects   - Medium: Covers main content but lacks detail in certain areas   - Low: Overly brief answers lacking necessary explanations or details |

**Reference**

1. Haoyu T, Kuo Y, Xin D, Chenxi Z, Mingwei Y, Hongyan W, et al. TCMLLM-PR: evaluation of large language models for prescription recommendation in traditional Chinese medicine. 2024;7(4):343-55.

2. 吉祥宇, 王鑫, 张鹤译, 孟昭鹏, 张俊华, 庄朋伟, et al. 面向中医药大模型的知识增强方法研究. 计算机科学与探索. 2024;18(10):2616-29.

3. 张鹤译, 王鑫, 韩立帆, 李钊, 陈子睿, 陈哲. 大语言模型融合知识图谱的问答系统研究. 计算机科学与探索. 2023;17(10):2377-88.

4. Hua R, Dong X, Wei Y, Shu Z, Yang P, Hu Y, et al. Lingdan: enhancing encoding of traditional Chinese medicine knowledge for clinical reasoning tasks with large language models. J Am Med Inform Assoc. 2024;31(9):2019-29.

5. Dai Y, Shao X, Zhang J, Chen Y, Chen Q, Liao J, et al. TCMChat: A generative large language model for traditional Chinese medicine. Pharmacol Res. 2024;210:107530.

6. Wei S, Peng X, Wang Y-f, Si J, Zhang W, Lu W, et al. BianCang: A Traditional Chinese Medicine Large Language Model. arXiv preprint arXiv:241111027. 2024.

7. Yu S, Xu X, Xu F, Li L. Enhancing the Traditional Chinese Medicine Capabilities of Large Language Model through Reinforcement Learning from AI Feedback. arXiv preprint arXiv:241100897. 2024.

8. Chen Y, Xiao Q, Yi J, Chen J, Wang M. Intelligent Understanding of Large Language Models in Traditional Chinese Medicine Based on Prompt Engineering Framework. arXiv preprint arXiv:241019451. 2024.

9. Yan Y, Ma T, Li R, Zheng X, Shan G, Li C. JingFang: A Traditional Chinese Medicine Large Language Model of Expert-Level Medical Diagnosis and Syndrome Differentiation-Based Treatment. arXiv preprint arXiv:250204345. 2025.

10. Zhang H, Wang X, Meng Z, Chen Z, Zhuang P, Jia Y, et al. Qibo: A large language model for traditional chinese medicine. arXiv preprint arXiv:240316056. 2024.

11. Zhou X, Dong X, Li C, Bai Y, Xu Y, Cheung KC, et al., editors. TCM-FTP: Fine-Tuning Large Language Models for Herbal Prescription Prediction2024: IEEE.

12. Zhou Z, Yang T, Hu K, editors. Traditional chinese medicine epidemic prevention and treatment question-answering model based on llms2023: IEEE.

13. Liu Y, Luo S, Zhong Z, Wu T, Zhang J, Ou P, et al. Hengqin-RA-v1: Advanced Large Language Model for Diagnosis and Treatment of Rheumatoid Arthritis with Dataset based Traditional Chinese Medicine. arXiv preprint arXiv:250102471. 2025.

14. Zhu J, Gong Q, Zhou C, Luan H. ZhongJing: A Locally Deployed Large Language Model for Traditional Chinese Medicine and Corresponding Evaluation Methodology: A Large Language Model for data fine-tuning in the field of Traditional Chinese Medicine, and a new evaluation method called TCMEval are proposed. Proceedings of the 2023 4th International Symposium on Artificial Intelligence for Medicine Science; Chengdu, China: Association for Computing Machinery; 2024. p. 1036–42.

15. Tan Y, Zhang Z, Li M, Pan F, Duan H, Huang Z, et al. MedChatZH: A tuning LLM for traditional Chinese medicine consultations. Comput Biol Med. 2024;172:108290.

16. Chin O, Jamil NS, Zainudin Z, Hitam NA, Ibrahim N, Sa'ahiry AHA, editors. OYEN: A User-Centric LLM-Based Bilingual Healthcare Chatbot. 2024 5th International Conference on Artificial Intelligence and Data Sciences (AiDAS); 2024 3-4 Sept. 2024.

17. Yang G, Liu X, Shi J, Wang Z, Wang G. TCM-GPT: Efficient pre-training of large language models for domain adaptation in Traditional Chinese Medicine. Computer Methods and Programs in Biomedicine Update. 2024;6:100158.

18. 海佳丽, 汪润, 袁良志, 张凯睿, 邓文萍, 肖勇, et al. 基于检索增强的中医药标准知识问答系统构建探索与实践. 数据分析与知识发现.1-13.

19. 张君冬, 杨松桦, 刘江峰, 黄奇. AIGC赋能中医古籍活化：Huang-Di大模型的构建. 图书馆论坛. 2024;44(10):103-12.

20. 张玉铭, 李红岩, 郎许锋, 周作建, 凌云, 王子琰. 基于检索增强生成技术的中医药问答大语言模型的构建. 南京中医药大学学报. 2024;40(12):1375-82.

21. Liu C, Sun K, Zhou Q, Duan Y, Shu J, Kan H, et al. CPMI-ChatGLM: parameter-efficient fine-tuning ChatGLM with Chinese patent medicine instructions. Sci Rep. 2024;14(1):6403.

22. Dou Y, Huang Y, Zhao X, Zou H, Shang J, Lu Y, et al. ShennongMGS: An LLM-based Chinese Medication Guidance System. 2024.

23. Wang Z, Li K, Ren Q, Yao K, Zhu Y, editors. Traditional Chinese Medicine Formula Classification Using Large Language Models. 2023 IEEE International Conference on Bioinformatics and Biomedicine (BIBM); 2023: IEEE.

24. Yue W, Wang X, Zhu W, Guan M, Zheng H, Wang P, et al. Tcmbench: A comprehensive benchmark for evaluating large language models in traditional chinese medicine. arXiv preprint arXiv:240601126. 2024.

25. Yu P, Song K, He F, Chen M, Lu J. TCMD: A Traditional Chinese Medicine QA Dataset for Evaluating Large Language Models. arXiv preprint arXiv:240604941. 2024.

26. Wang X, Chen GH, Song D, Zhang Z, Chen Z, Xiao Q, et al. Cmb: A comprehensive medical benchmark in chinese. arXiv preprint arXiv:230808833. 2023.

27. 曹露, 许林, 张宇洁, 张林帅, 付亚琴, 蒋涛. 大语言模型在中医领域的标准化评估. 南京中医药大学学报. 2024;40(12):1383-92.
